# Lung development genes, adult lung function and cognitive traits

**DOI:** 10.1093/braincomms/fcae380

**Published:** 2024-11-01

**Authors:** Mohammad Talaei, Sheena Waters, Laura Portas, Benjamin M Jacobs, James W Dodd, Charles R Marshall, Cosetta Minelli, Seif O Shaheen

**Affiliations:** Centre for Preventive Neurology, Wolfson Institute of Population Health, Queen Mary University of London, London EC1M 6BQ, UK; Centre for Preventive Neurology, Wolfson Institute of Population Health, Queen Mary University of London, London EC1M 6BQ, UK; Nuffield Department of Population Health, University of Oxford, Oxford OX3 7LF, UK; Big Data Institute, Li Ka Shing Centre for Health Information and Discovery, University of Oxford, Oxford OX3 7LF, UK; Centre for Preventive Neurology, Wolfson Institute of Population Health, Queen Mary University of London, London EC1M 6BQ, UK; MRC Integrative Epidemiology Unit (IEU), University of Bristol, Bristol BS8 2BN, UK; Academic Respiratory Unit, Southmead Hospital, University of Bristol, Bristol BS10 5NB, UK; Centre for Preventive Neurology, Wolfson Institute of Population Health, Queen Mary University of London, London EC1M 6BQ, UK; National Heart and Lung Institute, Imperial College London, London SW3 6LY, UK; Centre for Preventive Neurology, Wolfson Institute of Population Health, Queen Mary University of London, London EC1M 6BQ, UK; Allergy and Lung Health Unit, Melbourne School of Population and Global Health, The University of Melbourne, Melbourne, Victoria 3010, Australia

**Keywords:** lung function, cognitive function, dementia, genes, shared development

## Abstract

Lower lung function is associated with lower cognitive function and an increased risk of dementia. This has not been adequately explained and may partly reflect shared developmental pathways. In UK Biobank participants of European ancestry, we tested the association between lung function measures (forced vital capacity and forced expiratory volume in 1 s to forced vital capacity ratio; *n* = 306 476) and cognitive traits including nine cognitive function test scores (*n* = 32 321–428 609), all-cause dementia, Alzheimer’s disease and vascular dementia (6805, 2859 and 1544 cases, respectively, and ∼421 241 controls). In the same population, we derived summary statistics for associations between common genetic variants in 55 lung development genes and lung function measures and cognitive traits using adjusted linear/logistic regression models. Using a hypothesis-driven Bayesian co-localization analysis, we finally investigated the presence of shared genetic signals between lung function measures and cognitive traits at each of these 55 genes. Higher lung function measures were generally associated with higher scores of cognitive function tests as well as lower risk of dementia. The strongest association was between forced vital capacity and vascular dementia (adjusted hazard ratio 0.74 per standard deviation increase, 95% confidence interval 0.67–0.83). Of the 55 genes of interest, we found shared variants in four genes, namely: *CSNK2B* rs9267531 (forced vital capacity and forced expiratory volume in 1 s to forced vital capacity ratio with fluid intelligence and pairs matching), *NFATC3* rs548092276 & rs11275011 (forced expiratory volume in 1 s to forced vital capacity ratio with fluid intelligence), *PTCH1* rs2297086 & rs539078574 (forced expiratory volume in 1 s to forced vital capacity ratio with reaction time) and *KAT8* rs138259061 (forced vital capacity with pairs matching). However, the direction of effects was not in keeping with our hypothesis, i.e. variants associated with lower lung function were associated with better cognitive function or vice versa. We also found distinct variants associated with lung function and cognitive function in *KAT8* (forced vital capacity and Alzheimer’s disease) and *PTCH1* (forced vital capacity and forced expiratory volume in 1 s to forced vital capacity ratio with fluid intelligence and reaction time). The links between *CSNK2B* and *NFATC3* and cognitive traits have not been previously reported by genome-wide association studies. Despite shared genes and variants, our findings do not support the hypothesis that shared developmental signalling pathways explain the association of lower adult lung function with poorer cognitive function.

## Teaser

Talaei *et al*. found variants shared between adult lung function and cognitive traits in four lung development genes, but the direction of the effects was inconsistent within the pairs. Hence, these shared signals could not explain the association of lower adult lung function with poorer cognitive function and dementia.

## Graphical Abstract

**Graphical Abstract:**
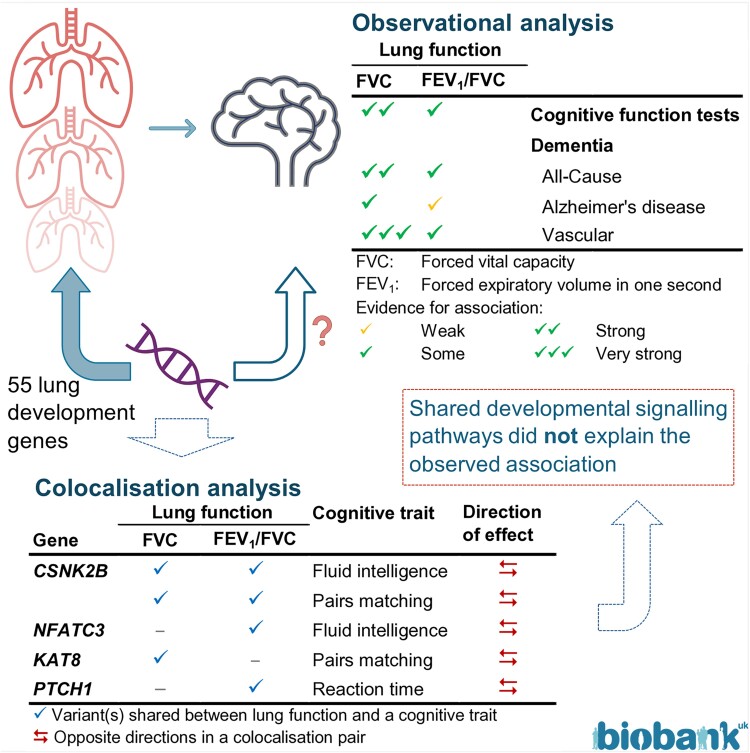

## Introduction

Lower lung function in adults is associated with lower cognitive function^1-5^ and a higher risk of dementia^5-9^ in epidemiological studies. A restrictive lung function impairment [reduced forced vital capacity (FVC)] is more strongly associated than obstructive impairment [reduced forced expiratory volume in 1 s (FEV_1_)/FVC ratio] with impaired cognitive function and dementia; the evidence is conflicting as to whether the link with dementia is stronger for vascular dementia (VD) than for Alzheimer’s disease.^7,8^ These associations are independent of many potential confounders, including cardiovascular risk factors, particularly smoking. Lung function is also associated with cognitive function in children.^10^ However, there is little evidence that lower lung function decreases cognitive function^11^ or increases the risk of Alzheimer’s disease.^12^ In the absence of a causal link between lung function and these cognitive traits, unknown shared risk factors are the most likely explanation for these associations.^11,12^ These shared risk factors could be genetic (suggested by twin studies^2,13^), environmental or both. Discovering common causes that influence both respiratory and neurological systems could help to explain comorbidity.

Shared risk factors are likely to operate from early in life. For instance, lower birthweight, a marker of poorer intra-uterine growth and development, is associated with both lower lung function^14^ and impaired cognitive function^15^ in adults. Lung function usually tracks from early childhood,^16^ so a lower developmental trajectory leads to a failure to attain maximal lung capacity as a young adult.^17^ Similarly, there is evidence that sub-optimal brain development may lead to impaired cognitive function in adult life through a failure to attain maximal organ size and functional capacity.^18,19^ Until recently, the ‘ageing’ paradigm has focused more on risk factors later in life influencing the decline in adult lung and cognitive function than on factors affecting differential reserve capacity. We propose that the association between lung function and cognitive traits in mid-late adulthood might be developmental and may partly reflect the signalling pathways shared by the lung and the brain.

In our previous work using a candidate gene approach in the UK Biobank (UKB), we identified 55 lung development genes associated with adult lung function, influencing both restrictive and obstructive patterns. Of these genes, 36 had not been previously identified in genome-wide association studies (GWASs).^20^ We hypothesize that these signalling pathways, which are critical to lung development and/or repair, may also be important for the development and repair of the brain. In this study, we first explored the association between lung function measures and various cognitive function tests and dementia. Our main aim was to investigate whether lung function and cognitive traits have shared lung development genes, and if so, whether they have shared or distinct variants within a locus.

## Materials and methods

We used the UKB data, a study of 502 543 volunteer participants aged 39–70, recruited from 22 study centres across United Kingdom (England, Scotland and Wales) in 2006–10. Data were collected on a large number of genetic and non-genetic risk factors for chronic disease and related traits at baseline. Sub-groups of participants were invited for repeat assessment visits at later stages.^21,22^ We used the UKB data for observational analysis (cross-sectionally and longitudinally) and genetic association analysis.

This study complies with the Declaration of Helsinki; the work was covered by the ethical approval for the UKB studies from the NHS National Research Ethics Service on 17 June 2011 (Ref 11/NW/0382) and extended on 18 June 2021 (Ref 21/NW/0157) with written informed consent obtained from all participants.

### Phenotypes

Lung function measures included FVC and FEV_1_/FVC at baseline (best measures^23^). Spirometry in the UKB was performed without bronchodilator administration, so only ‘pre-bronchodilator’ lung function is available.

Cognitive tests in the UKB were administered via a fully automated touchscreen interface and were described in detail online at http://biobank.ctsu.ox.ac.uk/crystal/label.cgi?id=100026. We included nine cognitive tests with a continuous outcome and a sufficient sample size (≥30 000). These were measured at two different assessment times, and for each test, we selected the time point with the highest sample size—i.e. four tests at baseline (Instance 0; 2006–10) and five tests at repeat assessment Visit 2 (Instance 2; from 2014). Almost all participants completed the pairs matching (visual declarative memory) and the reaction time (processing speed) tests at baseline. Sub-samples completed the numeric memory (working memory) and fluid intelligence (verbal and numerical reasoning) tests. At Visit 2 (on average 9.4 years after baseline), new tests were introduced, including trail making (Parts A and B; executive function), symbol digit substitution (processing speed), matrix pattern completion (non-verbal reasoning), tower rearranging (executive function) and paired associate learning (verbal declarative memory; further details in Supplementary Tables 1 and 2). Most of these tasks are computerized versions of well-validated cognitive tests,^24^ whereas the reasoning and reaction time tests were designed for the UKB. These cognitive tests corresponded well with their standardized and well-validated counterparts, exhibited good test–retest reliability^25^ and were valid measures of general cognitive functioning.^26^

All-cause dementia (ACD), Alzheimer’s disease and VD events were ascertained by combining linked medical records using the UKB’s algorithmically defined health outcomes, first occurrences of medical conditions and self-reports. See Supplementary Table 1 for more details about all phenotypes.

### Lung development genes

We previously identified 55 lung development genes associated with adult lung function in the UKB, including restrictive and obstructive patterns (Supplementary Table 2).^20^ In these genes, we analysed 15 298 variants with minor allele frequency ≥1% and imputation quality ≥0.5.

### Statistical analysis

The sample size varied according to the phenotypes analysed. We excluded participants who gave a self-reported ethnicity other than White, were related to another participant, had no genetic data, had a poor-quality genotype (outliers in heterozygosity and missing rates) or were already diagnosed with dementia at the time of the cognitive function assessments. After these exclusions, the sample size for candidate gene association analysis was 306 476 for lung function measures, ranged from 428 609 to 32 321 for cognitive function tests and was 6805 ACD cases (421 241 controls) including 2859 Alzheimer’s disease and 1544 VD cases (Supplementary Table 3).

We log-transformed pairs matching and reaction time scores due to severe skewness. Tests for which a higher value means worse function (pairs matching, reaction time and trail making) were scored such that a higher score indicates better performance (subtracted from the maximum). To make scores comparable, we calculated *Z*-scores for all cognitive function tests (difference from the mean divided by standard deviation).

To investigate a measure of general cognitive ability (g-factor), we estimated three latent variables using confirmatory factor analysis implemented in the Lavaan package in R.^27^ Two g-factors were estimated from the four cognitive function tests at baseline and the five cognitive function tests at Visit 2, which are measures of working memory or speed of processing (previously termed ‘executive function’^28^). A third g-factor was also estimated from all nine tests at Visit 2. Missing cognitive test data were imputed to generate g-factors using full information maximum likelihood, which gives unbiased parameter estimates and standard errors. All three models passed the key criteria for a good fit. The proportional variance explained and the loadings of the individual cognitive tests are presented in Supplementary Table 4.

We explored associations of FVC and FEV_1_/FVC (in quintiles and per standard deviation) as exposures, with cognitive function measures (*Z*-scores of nine individual tests and the three g-factors) as continuous outcomes using linear regression models and with dementia (ACD, Alzheimer’s disease and VD) as binary outcomes using Cox regression models. Follow-up time was from the date of the baseline examination until a dementia diagnosis, loss to follow-up, death or the recommended censoring dates at the time of analysis (31 March 2021 for England and Scotland, and 28 February 2018 for Wales). Covariates included age (years), sex, centre (22 categories), Townsend deprivation index at recruitment (continuous), 12 potentially modifiable risk factors for dementia^29^ including education (7 categories), hearing loss, traumatic brain injury, hypertension, alcohol >21 units per week, obesity, depression, social isolation, physical inactivity, air pollution, diabetes (all binary) and smoking at baseline (never, previous and current) and APOE4 alleles (3 categories). The analysis of FVC was additionally adjusted for height to ensure associations are independent of body size. To visualize the differences in dementia rates according to quintiles of lung function measures, cumulative hazard estimate plots were constructed after multivariable Cox regression models.

#### Summary statistics

**Figure 1:**
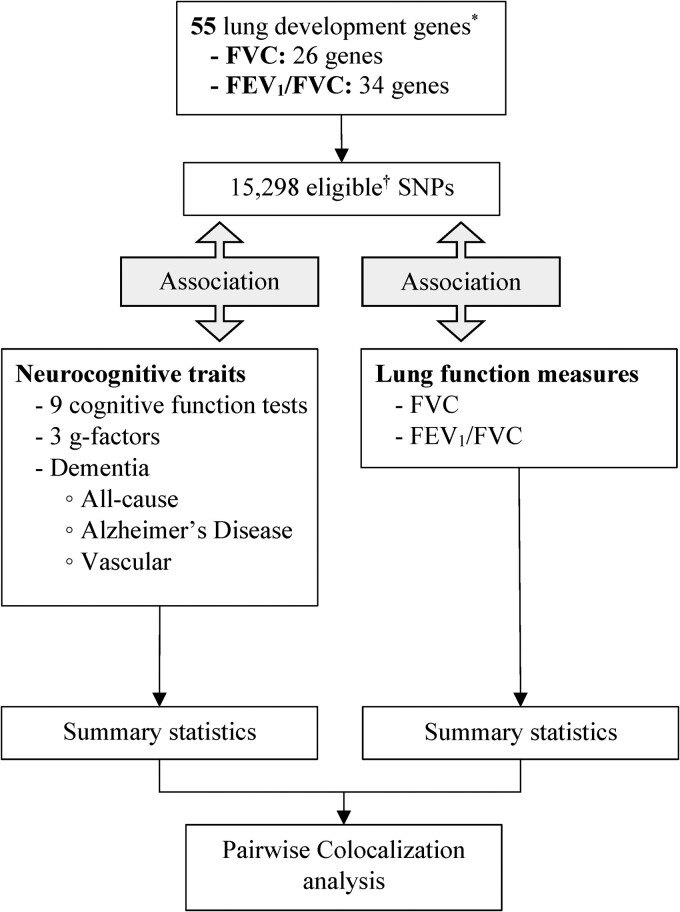
**Study profile for genetic analysis.** *Portas *et al*.^20^  ^†^Minor allele frequency of ≥1% and imputation quality ≥ 0.5. G-factor, general cognitive ability.

We have summarized our genetic analysis approach in Fig. 1. In our candidate gene association analysis, we calculated *β* coefficients, standard errors and *P*-values for the association of eligible variants within the 55 lung development genes with lung function measures, cognitive function tests, ACD, Alzheimer’s disease and VD using the Regenie program,^30^ assuming an additive genetic model and adjusting for age, sex, genotyping array, assessment centre, height (only for FVC) and the top 10 ancestry principal components.

We additionally used publicly available summary data for Alzheimer’s disease (https://www.ebi.ac.uk/gwas/publications/35379992) generated by a meta-analysis of a GWAS for clinically diagnosed cases from 15 European countries in the European Alzheimer and Dementia Biobank consortium (20 464 Alzheimer’s disease cases and 22 244 controls) and a proxy-Alzheimer’s disease and related dementia GWAS from the UKB dataset (using additive genetic models). The study involved 85 934 Alzheimer’s disease cases (39 106 clinically diagnosed Alzheimer’s disease and 46 828 proxy-Alzheimer’s disease and related dementia) and 401 577 controls.^31^ Of 15 298 variants in lung development genes, 12 598 single nucleotide polymorphisms (SNPs) could be matched with the GWAS meta-analysis dataset using variants' reference SNP (rs) numbers.

#### Co-localization analysis

Co-localization analysis assesses shared genetic aetiology across two traits to identify shared pathways.^32^ To explore whether the associations between lung function measures (Trait 1) and cognitive traits (Trait 2) were likely due to the same or distinct variants, we conducted pairwise co-localization analyses using a Bayesian statistical methodology (coloc.abf) implemented in the R package coloc (single-coloc).^32^ Assuming at most one causal variant per trait in each region (gene) and using effect estimates at each SNP, this algorithm calculates the support [posterior probability (PP)] for five mutually exclusive hypotheses: association with neither trait (PP.H0), with only one trait (PP.H1 and PP.H2), with both traits but distinct variants (PP.H3) and with both traits via shared single variants (PP.H4). We applied co-localization analysis to all eligible variants within each of the 55 genes. The default prior distributions implemented in coloc were modified to reflect the *a priori* knowledge of association of the genes (at least one SNP in each of the 55 genes) with a lung function measure, FVC and/or FEV_1_/FVC.

In cases with a high H4 or H3 reported by coloc (PP ≥0.50), we performed further investigation using a recently proposed Sum of Single Effects (SuSiE) regression framework for fine-mapping genetic signals. The SuSiE approach (coloc-SuSiE) allows simultaneous evaluation of evidence for association at multiple variants in proximity and provides a more accurate coloc inference.^33^ The linkage disequilibrium matrix needed for SuSiE analyses was derived from the same UKB genetic data. This hybrid approach utilizing single-coloc and coloc-SuSiE was shown to outperform other strategies to detect co-localization.^33^ In a sensitivity analysis, we tested co-localization using the default priors (*p*_1_ = *p*_2_ = 10^−4^; *p*_12_ = 10^−5^).

Further details about sample sizes, data processing, g-factors, genetic association analyses, modified priors and sensitivity analyses are explained in the online Supplementary material. To assess the novelty of our findings for cognitive traits, we searched published GWAS findings using GWAS Catalogue (www.ebi.ac.uk/gwas) and PhenoScanner on 5 July 2023.

This research utilized Queen Mary’s Apocrita high-performance computing facility, supported by QMUL Research-IT,^34^ through MobaXterm version v22.3 and OnDemand.^35^ Statistical analyses were carried out using Stata version 17.0 (StataCorp, College Station, TX, USA) and R version 4.2.2 (R Core Team, 2022) through RStudio (Rstudio Team, 2022), and for the genetic analyses, PLINK version 1.9-170906 and Regenie program version v2.2.4.^30^

## Results

Table 1 shows that participants with a higher FVC were younger, taller, more affluent, more likely to be men and with a higher level of education. Participants with a higher FEV_1_/FVC were also younger, more affluent and with a higher level of education, but more likely to be women and less likely to be current or ex-smoker. Cognitive function test scores were mildly correlated with each other (*r* < 0.37); g-factors were correlated with these scores mildly to strongly (*r* ranging from 0.31 to 0.91; Supplementary Fig. 1).

**Table 1:** Characteristics of participants according to quintiles of lung function measures

	Quintiles				
	Q1	Q2	Q3	Q4	Q5
**FVC**					
*N*	69 977	70 829	70 634	70 831	71 013
FVC best measure, L	2.54 ± 0.3	3.17 ± 0.1	3.66 ± 0.1	4.25 ± 0.2	5.26 ± 0.6
Age, years	60.5 ± 6.6	57.4 ± 7.5	55.9 ± 8.0	55.6 ± 8.1	53.2 ± 7.9
Men, *n* (%)	5634 (8.1)	11 464 (16.2)	26 712 (37.8)	51 765 (73.1)	67 710 (95.3)
Townsend deprivation index	−1.29 ± 3.1	−1.53 ± 2.9	−1.52 ± 2.9	−1.58 ± 2.9	−1.68 ± 2.9
Height (cm)	160 ± 6.4	164 ± 6.3	168 ± 6.6	173 ± 6.4	179 ± 6.3
Qualifications, *n* (%)					
College or university degree	15 955 (22.8)	21 475 (30.3)	24 169 (34.2)	25 606 (36.2)	29 255 (41.2)
A/AS levels	6903 (9.9)	8565 (12.1)	8636 (12.2)	8231 (11.6)	8581 (12.7)
O levels/GCSEs	16 310 (23.3)	16 538 (23.3)	15 365 (21.8)	14 195 (20.0)	14 104 (19.9)
CSEs/NVQ/HND/HNC or other	11 669 (16.7)	11 461 (16.2)	11 931 (16.9)	12 794 (18.1)	12 291 (17.3)
None of the above	19 140 (27.4)	12 790 (18.1)	10 533 (14.9)	10 005 (14.1)	6782 (9.6)
Smoking, *n* (%)					
Never	37 550 (53.9)	39 249 (55.6)	37 319 (53.0)	36 211 (51.3)	38 483 (54.3)
Previous	24 737 (35.5)	24 972 (35.4)	26 134 (37.1)	26 882 (38.1)	24 640 (35.9)
Current	7367 (10.6)	6376 (9.0)	6939 (9.9)	7534 (10.7)	7733 (10.9)
**FEV_1_/FVC**					
*N*	70 626	69 064	71 587	71 336	70 671
FEV_1_/FVC best measure	0.65 ± 0.1	0.73 ± 0.0	0.76 ± 0.0	0.79 ± 0.0	0.83 ± 0.0
Age, years	59.0 ± 7.5	57.7 ± 7.7	56.8 ± 7.8	55.6 ± 7.9	53.6 ± 8.0
Men, *n* (%)	38 502 (54.5)	32 373 (46.9)	31 355 (43.8)	30 213 (42.4)	30 842 (43.6)
Townsend deprivation index	−1.23 ± 3.1	−1.56 ± 2.9	−1.62 ± 2.9	−1.63 ± 2.9	−1.56 ± 2.9
Height (cm)	170 ± 9.4	169 ± 9.2	169 ± 9.2	168 ± 9.1	168 ± 9.1
Qualifications, *n* (%)					
College or university degree	20 903 (29.6)	22 509 (32.6)	23 812 (33.3)	24 393 (34.2)	24 843 (35.2)
A/AS levels	7294 (10.3)	7657 (11.1)	8487 (11.9)	8656 (12.1)	8822 (12.6)
O levels/GCSEs	14 244 (20.2)	14 841 (21.5)	15 701 (21.9)	15 748 (22.1)	15 978 (22.6)
CSEs/NVQ/HND/HNC or other	12 000 (17.0)	11 566 (16.7)	12 131 (16.9)	12 088 (16.9)	12 361 (17.5)
None of the above	16 185 (22.9)	12 491 (18.1)	11 456 (16.0)	10 451 (14.7)	8667 (12.3)
Smoking, *n* (%)					
Never	29 721 (42.3)	35 215 (51.1)	39 178 (54.9)	41 228 (58.0)	43 470 (61.7)
Previous	28 004 (39.8)	26 474 (38.4)	25 872 (36.3)	24 638 (34.6)	22 377 (32.8)
Current	12 590 (17.9)	7165 (10.4)	6309 (8.8)	5258 (7.4)	4627 (6.6)

A level, advanced level; AS, advanced subsidiary; GCSE, general certificate of secondary education; CSE, certificate of secondary education; NVQ, national vocational qualification; HND, higher national diploma; HNC, higher national certificates.

### Observational analyses

We found evidence of positive associations between all cognitive function tests and at least one of the two lung function measures. FVC was associated with seven cognitive function tests (all except pairs matching and tower rearranging) and all g-factors, with evidence of a dose–response for all and with the numeric memory test showing the strongest association, followed by the reaction time test (Table 2, expanded models in Supplementary Tables 5 and 6). FEV_1_/FVC was associated with five cognitive function tests and the two g-factors at Visit 2 (Table 3). Among the three tests associated with both lung function measures, reaction time and fluid intelligence were associated, respectively, six and four times more strongly with FVC than FEV_1_/FVC (per SD increase), while associations with each lung function parameter were similar for the trail making B-A test.

**Table 2:** Linear regression coefficients (95% confidence interval) for cognitive function test Z-scores according to FVC at baseline, adjusted for potential confounders

Median (IQR), L	Quintiles of FVC					*P*-trend	Per SD
	Q1	Q2	Q3	Q4	Q5		
	2.62 (2.37–2.78)	3.17 (3.05–3.29)	3.65 (3.53–3.78)	4.24 (4.08–4.42)	5.12 (4.84–5.53)		
Pairs matching	0.00	0.02 (0.00, 0.03)	0.01 (−0.00, 0.02)	0.01 (−0.00, 0.02)	0.02 (0.00, 0.03)	0.11	0.00 (−0.00, 0.01)
Reaction time	0.00	0.04 (0.03, 0.05)	0.07 (0.06, 0.08)	0.10 (0.09, 0.12)	0.14 (0.12, 0.15)	<0.001	0.05 (0.05, 0.06)
Fluid intelligence	0.00	0.04 (0.02, 0.05)	0.06 (0.04, 0.08)	0.09 (0.07, 0.11)	0.13 (0.10, 0.15)	<0.001	0.05 (0.04, 0.06)
Numeric memory	0.00	0.06 (0.03, 0.09)	0.10 (0.07, 0.14)	0.13 (0.09, 0.17)	0.20 (0.15, 0.25)	<0.001	0.08 (0.06, 0.10)
Trail making B-A	0.00	0.04 (0.00, 0.09)	0.08 (0.03, 0.12)	0.10 (0.05, 0.15)	0.12 (0.06, 0.18)	<0.001	0.03 (0.01, 0.05)
Symbol digit	0.00	0.01 (−0.03, 0.05)	0.02 (−0.02, 0.06)	0.05 (0.00, 0.10)	0.08 (0.03, 0.13)	0.001	0.04 (0.02, 0.06)
Matrix pattern	0.00	0.05 (0.01, 0.10)	0.03 (−0.01, 0.08)	0.08 (0.03, 0.13)	0.13 (0.07, 0.19)	<0.001	0.05 (0.03, 0.06)
Tower rearranging	0.00	0.00 (−0.04, 0.05)	0.02 (−0.02, 0.07)	0.04 (−0.01, 0.09)	0.04 (−0.02, 0.10)	0.18	0.01 (−0.01, 0.03)
Paired learning	0.00	0.05 (0.01, 0.09)	0.05 (0.01, 0.10)	0.11 (0.06, 0.16)	0.12 (0.07, 0.18)	<0.001	0.03 (0.01, 0.05)
G-factor							
All tests at baseline	0.00	0.06 (0.04, 0.08)	0.07 (0.04, 0.09)	0.09 (0.05, 0.12)	0.13 (0.09, 0.16)	<0.001	0.05 (0.03, 0.06)
EF at Visit 2	0.00	0.01 (−0.01, 0.04)	0.03 (0.00, 0.06)	0.06 (0.03, 0.09)	0.07 (0.04, 0.11)	<0.001	0.03 (0.02, 0.04)
All tests at Visit 2	0.00	0.04 (0.01, 0.07)	0.06 (0.03, 0.09)	0.10 (0.07, 0.14)	0.13 (0.09, 0.17)	<0.001	0.04 (0.03, 0.06)

The multivariable model included age, sex, assessment centre, Townsend deprivation index, 12 potentially modifiable risk factors for dementia including education, hearing loss, traumatic brain injury, hypertension, alcohol >21 units per week, obesity, smoking, depression, social isolation, physical inactivity, air pollution and diabetes and APOE4 alleles. Analysis of FVC was additionally adjusted for height. Pairs matching, reaction time, fluid intelligence, and numeric memory tests carried out at baseline were used. Trail making B-A, symbol digit, matrix pattern, tower rearranging and paired learning tests were carried out at Visit 2. Of participants included in genetic analyses, sample sizes with lung function measures ranged from 322 887 to 25 845.

EF, executive function (five components); g-factor, general cognitive ability; IQR, interquartile range.

**Table 3:** Linear regression coefficients (95% confidence interval) for cognitive function test Z-scores according to FEV_1_/FVC at baseline, adjusted for potential confounders

Median (IQR), L	Quintiles of FEV_1_/FVC					*P*-trend	Per SD
	Q1	Q2	Q3	Q4	Q5		
	0.68 (0.64–0.70)	0.73 (0.72–0.74)	0.76 (0.76–0.77)	0.79 (0.78–0.80)	0.82 (0.81–0.84)		
Pairs matching	0.00	0.00 (−0.01, 0.01)	0.01 (0.00, 0.02)	0.02 (0.01, 0.03)	0.02 (0.01, 0.03)	<0.001	0.01 (0.00, 0.01)
Reaction time	0.00	0.02 (0.01, 0.03)	0.02 (0.01, 0.03)	0.02 (0.01, 0.03)	0.02 (0.01, 0.03)	0.004	0.01 (0.01, 0.02)
Fluid intelligence	0.00	0.01 (−0.01, 0.03)	0.00 (−0.01, 0.02)	0.00 (−0.02, 0.02)	0.01 (−0.00, 0.03)	0.31	0.00 (−0.00, 0.01)
Numeric memory	0.00	−0.01 (−0.04, 0.02)	0.01 (−0.03, 0.04)	−0.01 (−0.04, 0.03)	0.01 (−0.03, 0.04)	0.78	0.00 (−0.01, 0.01)
Trail making B-A	0.00	0.05 (0.01, 0.09)	0.06 (0.02, 0.09)	0.06 (0.02, 0.10)	0.05 (0.01, 0.09)	0.004	0.03 (0.01, 0.04)
Symbol digit	0.00	0.04 (0.01, 0.08)	0.03 (−0.01, 0.06)	0.05 (0.02, 0.08)	0.04 (0.00, 0.07)	0.04	0.02 (0.00, 0.03)
Matrix pattern	0.00	0.03 (−0.00, 0.07)	0.03 (−0.01, 0.07)	0.03 (−0.01, 0.07)	0.01 (−0.03, 0.04)	0.66	0.01 (−0.01, 0.02)
Tower rearranging	0.00	0.02 (−0.02, 0.06)	0.00 (−0.04, 0.04)	0.06 (0.02, 0.10)	0.04 (0.00, 0.08)	0.007	0.02 (0.01, 0.04)
Paired learning	0.00	0.01 (−0.03, 0.05)	0.03 (−0.00, 0.07)	0.05 (0.01, 0.08)	0.03 (−0.01, 0.07)	0.02	0.01 (0.00, 0.03)
G-factor							
All tests at baseline	0.00	−0.01 (−0.03, 0.02)	0.00 (−0.02, 0.02)	−0.01 (−0.03, 0.02)	0.01 (−0.02, 0.03)	0.72	0.00 (−0.01, 0.01)
EF at Visit 2	0.00	0.04 (0.02, 0.06)	0.03 (0.01, 0.05)	0.05 (0.03, 0.07)	0.04 (0.02, 0.06)	<0.001	0.02 (0.01, 0.03)
All tests at Visit 2	0.00	0.03 (0.01, 0.06)	0.03 (0.00, 0.05)	0.04 (0.02, 0.07)	0.03 (0.01, 0.06)	0.004	0.02 (0.01, 0.03)

The multivariable model included age, sex, assessment centre, Townsend deprivation index, 12 potentially modifiable risk factors for dementia including education, hearing loss, traumatic brain injury, hypertension, alcohol >21 units per week, obesity, smoking, depression, social isolation, physical inactivity, air pollution and diabetes and APOE4 alleles. Pairs matching, reaction time, fluid intelligence and numeric memory tests carried out at baseline were used. Trail making B-A, symbol digit, matrix pattern, tower rearranging and paired learning tests were carried out at Visit 2. Of participants included in genetic analyses, sample sizes with lung function measures ranged from 322 887 to 25 845.

EF, executive function (five components); g-factor, general cognitive ability; IQR, interquartile range.

**Figure 2:**
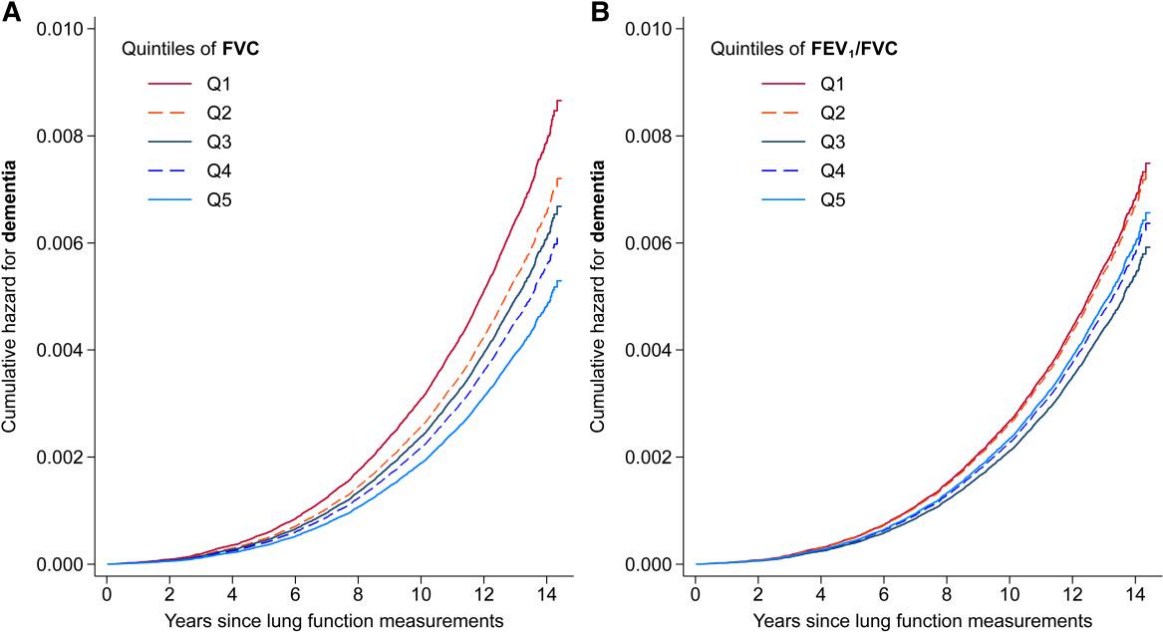
**Dementia cumulative hazard according to lung function measures.** Cumulative hazard of ACD according to quintiles of FVC (**A**) and FEV_1_/FVC (**B**) at baseline after controlling for potential confounders (*n* = 320 523 for both graphs).

During a median follow-up of 12.5 years, 4294 cases of incident ACD were recorded in participants included in this analysis (eligible for genetic analyses and with data on lung function), including 1855 cases of Alzheimer’s disease and 947 cases of VD. Figure 2 shows the adjusted cumulative hazard estimates for ACD over time according to quintiles of lung function measures. There was strong evidence of an inverse association between FVC and ACD, with evidence of a dose–response (Table 4, expanded models in Supplementary Table 7). We found evidence of a stronger inverse association for VD, while it was relatively weaker for Alzheimer’s disease. There was also weak evidence of an inverse association between FEV_1_/FVC and ACD, particularly VD.

**Table 4:** Hazard ratio (95% confidence interval) for incident dementia according to lung function measures at baseline, adjusted for potential confounders

	Quintiles of lung function measures					*P*-trend	Per SD
	Q1	Q2	Q3	Q4	Q5		
**FVC**							
ACD							
Cases/person-years	1396/746 473	861/780 074	793/784 443	785/790 132	459/803 555		
Fully adjusted model	1.00	0.83 (0.76–0.91)	0.77 (0.70–0.86)	0.71 (0.63–0.80)	0.61 (0.53–0.71)	<0.001	0.82 (0.78–0.86)
VD							
Cases/person-years	318/746 473	197/780 073	173/784 443	166/790 132	93/803 555		
Fully adjusted model	1.00	0.81 (0.67–0.98)	0.64 (0.51–0.79)	0.56 (0.44–0.72)	0.52 (0.39–0.71)	<0.001	0.74 (0.67–0.83)
Alzheimer’s disease							
Cases/person-years	640/746 473	374/780 074	311/784 443	319/790 132	211/803 555		
Fully adjusted model	1.00	0.84 (0.73–0.96)	0.77 (0.65–0.90)	0.76 (0.63–0.92)	0.78 (0.63–0.97)	0.02	0.87 (0.81–0.95)
**FEV_1_/FVC**							
ACD							
Cases/person-years	1284/761 443	1017/764 272	756/796 038	694/795 347	543/787 577		
Fully adjusted model	1.00	0.98 (0.90–1.07)	0.80 (0.73–0.88)	0.86 (0.79–0.95)	0.90 (0.81–1.00)	<0.001	0.92 (0.89–0.94)
VD							
Cases/person-years	323/765 511	224/767 438	162/799 602	145/798 561	104/791 057		
Fully adjusted model	1.00	0.91 (0.77–1.08)	0.72 (0.60–0.88)	0.74 (0.61–0.91)	0.74 (0.59–0.93)	<0.001	0.89 (0.84–0.94)
Alzheimer’s disease							
Cases/person-years	535/761 443	466/764 272	335/796 038	284/795 347	235/787 577		
Fully adjusted model	1.00	1.07 (0.94–1.21)	0.85 (0.74–0.97)	0.86 (0.74–1.00)	0.97 (0.83–1.14)	0.07	0.93 (0.89–0.97)

The multivariable model included age, sex, assessment centre, Townsend deprivation index, 12 potentially modifiable risk factors for dementia including education, hearing loss, traumatic brain injury, hypertension, alcohol >21 units per week, obesity, smoking, depression, social isolation, physical inactivity, air pollution and diabetes and APOE4 alleles. Analysis of FVC was additionally adjusted for height. Of participants included in genetic analyses, after excluding 120 participants with dementia at baseline, 4337 cases of incident ACD were included in these analyses, including 1882 cases of incident Alzheimer’s disease and 958 cases of VD.

### Co-localization analyses

Of the 55 genes linked to lung development, the same variant (or variants in high linkage disequilibrium) was shared between lung function measures and at least one cognitive trait in four genes indicating co-localization (high PP.H4), namely: *CSNK2B* with FVC and FEV_1_/FVC for fluid intelligence and pairs matching, *NFATC3* with FEV_1_/FVC for fluid intelligence, *PTCH1* with FEV_1_/FVC for reaction time and *KAT8* with FVC for pairs matching (Table 5). We also found distinct variants (high PP.H3) in *KAT8* with FVC for Alzheimer’s disease (from GWAS meta-analysis), in *PTCH1* with FVC and FEV_1_/FVC for fluid intelligence and reaction time and in *SERPINC1* with FVC for reaction time.

**Table 5:** Co-localization results for lung function measures and cognitive traits

Gene	Lung function trait	Cognitive trait	Highest PP	SNP	Linkage disequilibrium (*R*^2^)
*CSNK2B*	FEV_1_/FVC	Fluid intelligence	H4: 0.976	**rs9267531**	
		Pairs matching	H4: 0.911	**rs9267531**	
	FVC	Fluid intelligence	H4: 0.995^a^	**rs9267531**	
			H3: 0.948^a^	rs3117578 & rs9267531	0.02
		Pairs matching	H4: 0.946^a^	**rs9267531**	
			H3: 0.941^a^	rs3117578 & rs9267531	0.02
*NFATC3*	FEV_1_/FVC	Fluid intelligence	H4: 0.802^a^	**rs548092276 & rs11275011**	0.85^b^
*ITGAV*	FEV_1_/FVC	Pairs matching	H4: 0.678	rs2084448^c^	
*KAT8*	FVC	Pairs matching	H4: 0.829	**rs138259061**	
		Alzheimer’s disease^d^	H3: 0.989	rs1978487, rs11865499^e^	0.23
*PTCH1*	FVC	Reaction time	H3: 0.960^a^	rs113154802, rs539078574	0.13
		Fluid intelligence	H3: 0.999^a^	rs113154802, rs28496034	0.19
	FEV_1_/FVC	Reaction time	H4: 0.527^a^	**rs2297086 & rs539078574**	0.54
			H3: 0.960^a^	rs75614054, rs539078574	0.13
		Fluid intelligence	H3: 0.601^a^	rs2297086, rs28496034	0.86
			H3: 0.999^a^	rs75614054, rs28496034	0.19
*SERPINC1*	FVC	Reaction time	H3: 0.612^a^	rs2227603, rs2227592	0.003

Highlighted in bold are variants with evidence for co-localization where the variants for each trait in the co-localization pair were either the same (a high gene PP.H4 and a high SNP PP.H4: variants in *CSNK2B1* and *KAT8*) or distinct but with correlated signals (high SNP PP.H4 for the pair by coloc-SuSiE: variants in *NFATC3* and *PTCH1* separated by ‘&’).

^a^By SuSiE-coloc.

^b^Variants rs548092276 and rs11275011 are not in 1000G reference panel (GRCh37 and GRCh38), so *R*^2^ is reported based on data for the two variants in UKB.

^c^SNP PP.H4 = 18.8% (the highest reported for variants in *ITGAV*), not large enough to form a co-localization case.

^d^From GWAS meta-analysis.

^e^Variants detected by fine mapping (the highest PP) as SuSiE did not operate for *KAT8*.

In a sensitivity analysis using the default prior probabilities, PP.H4 values were slightly increased and PP.H3 values slightly attenuated (Supplementary Table 8). However, the findings were overall similar, apart from two new genes with co-localization being identified (*MMP24* for FVC and symbol digit substitution; *TNS1* for FVC and VD), and one gene with distinct variants being lost (*SERPINC1* for FVC and reaction time).

Among the variants showing evidence of co-localization, those associated with lower lung function (rs548092276, rs11275011 and rs138259061) were associated with higher cognitive function. In contrast, variants associated with higher lung function (rs9267531, rs2297086, rs539078574, rs28496034 and rs6120880) were associated with lower cognitive function (see Beta coefficients, Table 6). The only exception was *TNS1*-rs2571445 (from sensitivity analysis), associated with a lower FEV_1_/FVC and a higher risk of VD (Supplementary Table 9).

**Table 6:** Characteristics of variants and their effects where the lung function measure co-localized with cognitive traits (bold variants) or distinct variants were associated with each trait

Gene	ID	Effect allele	MAF	Lung function			Cognitive tests		
				Trait	Beta	*P*	Trait	Beta	*P*
*CSNK2B*	**rs9267531**	A	0.127	FEV_1_/FVC	0.295	5.59E−38	Fluid intelligence	−0.023	9.28E−06
							Pairs matching	−0.013	1.33E−05
				FVC	11.5	9.58E−08			
	rs3117578	A	0.146	FVC	17.8	8.16E−18	^d^	^d^	^d^
*NFATC3*	**rs548092276** ^a^	C	0.164	FEV_1_/FVC	−0.142	4.99E−11	Fluid intelligence	0.019	9.25E−05
	**rs11275011** ^a^	T	0.165	FEV_1_/FVC	−0.135	2.42E−10	Fluid intelligence	0.022	4.69E−06
*ITGAV*	rs2084448	T	0.294	FEV_1_/FVC	0.134	5.68E−15	Pairs matching	−0.009	5.59E−05
*KAT8*	**rs138259061**	A	0.363	FVC	−9.555	5.34E−10	Pairs matching	0.012	1.19E−07
	rs1978487	C	0.366	FVC	9.29	1.41E−09	^d^	^d^	^d^
	rs11865499	A	0.298	^d^	^d^	^d^	Alzheimer’s disease	0.053^e^	3.83E−09
*PTCH1*	rs113154802	C	0.089	FVC	−24.42	1.76E−21	^d^	^d^	^d^
	rs539078574	AT	0.407	^d^	^d^	^d^	Reaction time	−0.010	1.21E−06
	rs28496034	C	0.330	^d^	^d^	^d^	Fluid intelligence	−0.024	2.08E−10
	**rs2297086** ^b,c^	G	0.360	FEV_1_/FVC	0.180	1.86E−28	Reaction time	−0.008	3.93E−05
							Fluid intelligence	−0.021	1.50E−08
	**rs539078574** ^b^	AT	0.407	FEV_1_/FVC	0.130	1.43E−15	Reaction time	−0.010	1.21E−06
	rs75614054	C	0.086	FEV_1_/FVC	0.341	8.16E−36	^d^	^d^	^d^
	**rs28496034** ^c^	C	0.330	FEV_1_/FVC	0.174	1.76E−25	Fluid intelligence	−0.024	2.08E−10
*SERPINC1*	rs2227603	A	0.028	FVC	−22.7	3.67E−07	^d^	^d^	^d^
	rs2227592	C	0.104	^d^	^d^	^d^	Reaction time	0.013	6.12E−05

SNPs effects and their directions are based on the association analyses done by Regenie. Strong cases of co-localization at the variant level are bold (indicating a high gene and SNP H4.PP).

^a,b,c^Pairs of variants with co-localization by SuSiE-coloc.

^d^‘Not applicable’ because in cases with a high gene PP.H3 (non-bold variants), the variant is only linked to one of the outcomes.

^e^OR (95% confidence interval): 1.05 (1.04–1.07). MAF, minor allele frequency.

For the genes with shared signals for lung function and cognitive function, we investigated gene expression in both human lung and brain tissues, using the ‘Expression Atlas’ database (https://www.ebi.ac.uk/gxa/home#) for RNA expression (mainly based on Genotype-Tissue Expression Project, GTEx^36^) and ‘The Human Protein Atlas’ (www.proteinatlas.org) for protein expression. This showed that all these genes are expressed in the human lung and brain, while protein expression was also reported for *CSNK2B*, *PTCH1*, *ITGAV* and *TNS1* (Supplementary Table 10).

## Discussion

### Observational findings

In UKB participants, higher lung function measures were associated with higher scores of cognitive function tests and lower risk of ACD, Alzheimer’s disease and VD. FVC was associated with more cognitive function tests, and more strongly, than was FEV_1_/FVC. The FVC association with VD was much stronger than with ACD and Alzheimer’s disease and stronger than associations for FEV_1_/FVC.

Our findings are in line with previous studies that showed FVC is more strongly associated than FEV_1_/FVC with dementia,^5-7^ and lung function, particularly FVC, is more strongly associated with VD than with Alzheimer’s disease.^6-8^ The latter observation is in keeping with the causal link between FVC and cardiovascular disease.^37^ We expanded those findings in the large UKB study by using multiple cognitive function tests and by analysing lung function measures as continuous outcomes rather than binary definitions of obstructive and restrictive impairment as used in a previous UKB analysis.^7^ Compared with previous studies, which investigated the link between lung function and cognitive function,^1-5^ our analysis was more comprehensive, clarifying the extent of the relationships of cognitive function with lung function, particularly FVC. Of note, the cognitive function tests assessed rather specific aspects of cognitive function, which were only mildly correlated.

### Co-localization findings

Of the 55 lung development genes, there were shared variants between lung function measures and cognitive traits (7 colocalization pairs) in 4 genes. However, the direction of effects was inconsistent for the two traits in the co-localization pair. There were also additional signals, but with distinct variants.

To our knowledge, this is the first study to investigate shared developmental pathways for lung function and cognitive traits. These genes (with shared or distinct variants) are all protein coding and involved in a range of functions, including transcriptional regulation (*CSNK2B*, *NFATC3* and *KAT8*), extracellular matrix (*ITGAV*, *MMP24* and *TNS1*), growth factors (*PTCH1*) and cell-to-cell adhesion and cytoskeleton (*TNS1*). All these genes are expressed in both the lung and the brain, and protein expression was detected for many, indicating their functional consequences.

The strongest co-localization signals were for the *CSNK2B* and *KAT8* genes. *CSNK2B* encodes for the regulatory subunit of casein kinase II, a protein that regulates the Wnt signalling pathways, known to orchestrate diverse cellular processes, particularly during development.^38^ These pathways have key roles in the development of the lung (segregation of the airway and alveolar compartments, etc.)^39^ and the nervous system (synaptic plasticity, neuronal survival, neurogenesis, etc.).^40,41^ In mouse models, *CSNK2B* regulates the proliferation and differentiation of neural stem cells, regulates the morphology of neurons and modulates synaptic transmission.^42^  *De novo* missense variants of *CSNK2B* cause an intellectual disability syndrome.^38^ In keeping with our finding, but through a different variant, *CSNK2B* was moderately associated with working and long-term memory in healthy young Chinese participants.^42^

*KAT8* is also involved in mechanisms in lung and brain. It encodes for lysine acetyltransferase 8, an essential enzyme for the acetylation of histone H4 at lysine 16 (H4K16), and is critical for DNA damage responses and nuclear architecture. It was one of the novel genes associated with FVC in UKB, which was replicated in external populations,^20^ though details about its actual function in the lung are yet to be explored. The importance of *KAT8* in neural stem and progenitor cell development, and thus cerebral development, has been shown in mouse models and through its link with syndromic intellectual disability disorders.^43^ In Alzheimer’s disease, H4K16 is substantially lost compared with normal ageing. Furthermore, a local genetic correlation between Alzheimer’s disease and cerebellar volume was found in a locus that includes the Alzheimer’s disease’s lead SNP in *KAT8*, which further illustrates this gene’s key role in neurodegenerative disorders.^44^

Despite the strong co-localization findings, at least for *CSNK2B* and *KAT8* with plausible biological effects, the direction of effects was mostly not consistent with our hypothesis that a variant that enhances lung function also improves cognitive function score. However, the paradoxical direction of effect is not unprecedented, even between different measures of a phenotype. In a cognitive function GWAS, almost 29% of variants for general cognitive function had an opposite direction of effect with reaction time (a component of general cognitive function), whereas the direction of effect for 47% of variants associated with reaction time was the opposite of that for general cognitive function.^45^ Nevertheless, while our findings support shared developmental origins to some extent, they do not explain the observational associations between lung function and neurocognitive traits.

While there is limited evidence for genetic correlations between cognitive phenotypes and FEV_1_ using an agnostic GWAS approach in the UKB,^46^ recent Mendelian randomization studies did not support a causal link between lung function and general cognitive function^11^ or Alzheimer’s disease.^12^ Therefore, the observed link between lung function and cognitive traits may be explained by unknown developmental pathways, other genetic pathways, shared environmental risk factors or gene–environment interactions. Future studies exploring environmental factors need to investigate novel factors, other than those many potential confounders for which these associations were already controlled for, and to consider optimizing assessments of known confounders to minimize residual confounding.

### Strengths and limitations

The various cognitive function tests carried out by the UKB enabled us to investigate diverse aspects of cognition, in addition to dementia, which is at the end of the clinical spectrum. We also used g-factors to test the global cognitive function ability of our participants. Three of the genes found in our co-localization analysis (*CSNK2B*, *ITGAV* and *NFATC3*) have not been previously identified as being associated with any cognitive trait in GWASs. This discovery gives added value to our study and highlights the potential of this type of hypothesis-driven investigation to complement an agnostic GWAS approach for the identification of novel candidate genes, although these findings need to be replicated. The large sample size provided by the UKB, complemented by meta-analyses of GWASs for Alzheimer’s disease, meant we had substantial statistical power to analyse cognitive phenotypes. However, the co-localization signals were mainly with cognitive function tests at baseline (reaction time, fluid intelligence and pairs matching) and with the number of participants larger than six figures—much higher than those at Visit 2. In GWASs, it is known that many variants for complex phenotypes are found when the sample size increase to the six-figure level.^47,48^ This raises the possibility of further shared signals being detected in the future once sample sizes increase for some outcomes in the UKB (e.g. accumulation of VD cases and completion of ongoing cognitive function assessments) or a meta-analysis of GWASs for VD becomes available. The sample sizes, which enable identification of all causal variants, are still out of reach, with an additional limitation for rarer variants or those in regions of particular allelic heterogeneity, even with sophisticated methods such as SuSiE.^33^ Given the age at which lung function and cognitive phenotypes were measured in the UKB participants, we cannot say whether our findings predominantly reflect the effects of these genes on development or repair. For example, a lower level of lung function in late adulthood may have arisen through sub-optimal growth and/or accelerated decline. Various accuracy measures were estimated for different types of dementia in the UKB, with VD having the lowest positive predictive value;^49^ hence, the potential biases inherent in using these data need to be considered. Finally, healthy volunteer bias is well documented in the UKB,^50^ and our analyses were limited to participants of White ethnic background, which may restrict the generalizability of our findings.

## Conclusion

Although we found evidence for shared genetic signals, our findings do not support the hypothesis that shared developmental signalling pathways explain the observed association of lower adult lung function with poorer cognitive function or higher risk of dementia.

## Supplementary Material

fcae380_Supplementary_Data

## Data Availability

This research was conducted using the UK Biobank resource under access application 78867. The UK Biobank will make the data available to all bona fide researchers for all types of health-related research that is in the public interest, without preferential or exclusive access for any persons. All researchers will be subject to the same application process and approval criteria as specified by the UK Biobank. For more details on the access procedure, see the UK Biobank website: http://www.ukbiobank.ac.uk/register-apply/. Scripts are available at https://github.com/mtalaei/DevOr.git.

## References

[1] Chyou P-H, White LR, Yano K, et al Pulmonary function measures as predictors and correlates of cognitive functioning in later life. Am J Epidemiol. 1996;143(8):750–756. 8610684 10.1093/oxfordjournals.aje.a008812

[2] Emery CF, Pedersen NL, Svartengren M, McClearn GE. Longitudinal and genetic effects in the relationship between pulmonary function and cognitive performance. J Gerontol B Psychol Sci Soc Sci. 1998;53B(5):P311–P3117. 10.1093/geronb/53b.5.p311 9750568

[3] Richards M, Strachan D, Hardy R, Kuh D, Wadsworth M. Lung function and cognitive ability in a longitudinal birth cohort study. Psychosom Med. 2005;67(4):602–608. 16046374 10.1097/01.psy.0000170337.51848.68

[4] Vidal J-S, Aspelund T, Jonsdottir MK, et al Pulmonary function impairment may be an early risk factor for late-life cognitive impairment. J Am Geriatr Soc. 2013;61(1):79–83. 23311554 10.1111/jgs.12069 PMC3545414

[5] Pathan SS, Gottesman RF, Mosley TH, Knopman DS, Sharrett AR, Alonso A. Association of lung function with cognitive decline and dementia: The Atherosclerosis Risk in Communities (ARIC) study. Eur J Neurol. 2011;18(6):888–898. 21244584 10.1111/j.1468-1331.2010.03340.x PMC3092022

[6] Xiao T, Wijnant SRA, Licher S, et al Lung function impairment and the risk of incident dementia: The Rotterdam study. J Alzheimers Dis. 2021;82(2):621–630. 34057085 10.3233/JAD-210162 PMC8385522

[7] Zhou L, Yang H, Zhang Y, et al Association of impaired lung function with dementia, and brain magnetic resonance imaging indices: A large population-based longitudinal study. Age Ageing. 2022;51(11):afac269. 36413587 10.1093/ageing/afac269

[8] Lutsey PL, Chen N, Mirabelli MC, et al Impaired lung function, lung disease, and risk of incident dementia. Am J Respir Crit Care Med. 2019;199(11):1385–1396. 30433810 10.1164/rccm.201807-1220OC PMC6543713

[9] Li Q-Y, Li X-M, Hu H-Y, et al Associations of lung function decline with risks of cognitive impairment and dementia: A meta-analysis and systematic review. J Alzheimers Dis. 2023;92(3):853–873. 36806509 10.3233/JAD-221136

[10] Grenville J, Granell R, Dodd J. Lung function and cognitive ability in children: A UK birth cohort study. BMJ Open Respir Res. 2023;10(1):e001528. 10.1136/bmjresp-2022-001528 PMC10163472 37130649

[11] Higbee DH, Granell R, Hemani G, Smith GD, Dodd JW. Lung function, COPD and cognitive function: A multivariable and two sample Mendelian randomization study. BMC Pulm Med. 2021;21(1):246. 34294062 10.1186/s12890-021-01611-6 PMC8296721

[12] Higbee D, Granell R, Walton E, Korologou-Linden R, Davey Smith G, Dodd J. Examining the possible causal relationship between lung function, COPD and Alzheimer's disease: A Mendelian randomisation study. BMJ Open Respir Res. 2021;8(1):e000759. 10.1136/bmjresp-2020-000759 PMC8264898 34233891

[13] Finkel D, Reynolds CA, Emery CF, Pedersen NL. Genetic and environmental variation in lung function drives subsequent variation in aging of fluid intelligence. Behav Genet. 2013;43(4):274–285. 23760789 10.1007/s10519-013-9600-3 PMC3753107

[14] Saad NJ, Patel J, Burney P, Minelli C. Birth weight and lung function in adulthood: A systematic review and meta-analysis. Ann Am Thorac Soc. 2017;14(6):994–1004. 28362513 10.1513/AnnalsATS.201609-746SR

[15] Grove BJ, Lim SJ, Gale CR, Shenkin SD. Birth weight and cognitive ability in adulthood: A systematic review and meta-analysis. Intelligence. 2017;61:146–158.

[16] Martinez FD . Early-Life origins of chronic obstructive pulmonary disease. N Engl J Med. 2016;375(9):871–878. 27579637 10.1056/NEJMra1603287

[17] Belgrave DCM, Granell R, Turner SW, et al Lung function trajectories from pre-school age to adulthood and their associations with early life factors: A retrospective analysis of three population-based birth cohort studies. Lancet Respir Med. 2018;6(7):526–534. 29628377 10.1016/S2213-2600(18)30099-7

[18] Walhovd KB, Fjell AM, Brown TT, et al Long-term influence of normal variation in neonatal characteristics on human brain development. Proc Natl Acad Sci U S A. 2012;109(49):20089–20094. 23169628 10.1073/pnas.1208180109 PMC3523836

[19] Muller M, Sigurdsson S, Kjartansson O, et al Birth size and brain function 75 years later. Pediatrics. 2014;134(4):761–770. 25180277 10.1542/peds.2014-1108 PMC4179101

[20] Portas L, Pereira M, Shaheen SO, et al Lung development genes and adult lung function. Am J Respir Crit Care Med. 2020;202(6):853–865. 32392078 10.1164/rccm.201912-2338OC PMC7491406

[21] Bycroft C, Freeman C, Petkova D, et al The UK Biobank resource with deep phenotyping and genomic data. Nature. 2018;562(7726):203–209. 30305743 10.1038/s41586-018-0579-z PMC6786975

[22] Sudlow C, Gallacher J, Allen N, et al UK biobank: An open access resource for identifying the causes of a wide range of complex diseases of middle and old age. PLoS Med. 2015;12(3):e1001779. 25826379 10.1371/journal.pmed.1001779 PMC4380465

[23] Wain LV, Shrine N, Miller S, et al Novel insights into the genetics of smoking behaviour, lung function, and chronic obstructive pulmonary disease (UK BiLEVE): A genetic association study in UK biobank. Lancet Respir Med. 2015;3(10):769–781. 26423011 10.1016/S2213-2600(15)00283-0 PMC4593935

[24] Gualtieri CT, Johnson LG. Reliability and validity of a computerized neurocognitive test battery, CNS vital signs. Arch Clin Neuropsychol. 2006;21(7):623–643. 17014981 10.1016/j.acn.2006.05.007

[25] Fawns-Ritchie C, Deary IJ. Reliability and validity of the UK Biobank cognitive tests. PLoS One. 2020;15(4):e0231627. 32310977 10.1371/journal.pone.0231627 PMC7170235

[26] Lyall DM, Cullen B, Allerhand M, et al Cognitive test scores in UK Biobank: Data reduction in 480,416 participants and longitudinal stability in 20,346 participants. PLoS One. 2016;11(4):e0154222. 27110937 10.1371/journal.pone.0154222 PMC4844168

[27] Rosseel Y . **Lavaan**: An *R* package for structural equation modeling. J Stat Softw. 2012;48(2):1–36.

[28] Tai XY, Chen C, Manohar S, Husain M. Impact of sleep duration on executive function and brain structure. Commun Biol. 2022;5(1):201. 35241774 10.1038/s42003-022-03123-3 PMC8894343

[29] Livingston G, Huntley J, Sommerlad A, et al Dementia prevention, intervention, and care: 2020 report of the Lancet Commission. Lancet. 2020;396(10248):413–446. 32738937 10.1016/S0140-6736(20)30367-6 PMC7392084

[30] Mbatchou J, Barnard L, Backman J, et al Computationally efficient whole-genome regression for quantitative and binary traits. Nat Genet. 2021;53(7):1097–1103. 34017140 10.1038/s41588-021-00870-7

[31] Bellenguez C, Kucukali F, Jansen IE, et al New insights into the genetic etiology of Alzheimer's disease and related dementias. Nat Genet. 2022;54(4):412–436. 35379992 10.1038/s41588-022-01024-z PMC9005347

[32] Giambartolomei C, Vukcevic D, Schadt EE, et al Bayesian test for colocalisation between pairs of genetic association studies using summary statistics. PLoS Genet. 2014;10(5):e1004383. 24830394 10.1371/journal.pgen.1004383 PMC4022491

[33] Wallace C . A more accurate method for colocalisation analysis allowing for multiple causal variants. PLoS Genet. 2021;17(9):e1009440. 34587156 10.1371/journal.pgen.1009440 PMC8504726

[34] Butcher S, King T, Zalewski L. High Performance Computing Cluster for Queen Mary University of London. Zenodo; 2017. 10.5281/zenodo.438045

[35] Hudak D, Johnson D, Chalker A, et al Open OnDemand: A web-based client portal for HPC centers. J Open Source Softw. 2018;3(25):622.

[36] The GTEx Consortium, Ardlie KG, Deluca DS, et al Human genomics. The genotype-tissue expression (GTEx) pilot analysis: Multitissue gene regulation in humans. Science. 2015;348(6235):648–660. 25954001 10.1126/science.1262110 PMC4547484

[37] Higbee DH, Granell R, Sanderson E, Davey Smith G, Dodd JW. Lung function and cardiovascular disease: A two-sample Mendelian randomisation study. Eur Respir J. 2021;58(3):2003196. 33574079 10.1183/13993003.03196-2020

[38] Asif M, Kaygusuz E, Shinawi M, et al *De novo* variants of *CSNK2B* cause a new intellectual disability-craniodigital syndrome by disrupting the canonical Wnt signaling pathway. HGG Adv. 2022;3(3):100111. 35571680 10.1016/j.xhgg.2022.100111 PMC9092267

[39] De Langhe SP, Reynolds SD. Wnt signaling in lung organogenesis. Organogenesis. 2008;4(2):100–108. 19279721 10.4161/org.4.2.5856 PMC2634255

[40] Palomer E, Buechler J, Salinas PC. Wnt signaling deregulation in the aging and Alzheimer’s brain. Front Cell Neurosci. 2019;13:227. 31191253 10.3389/fncel.2019.00227 PMC6538920

[41] Jia L, Pina-Crespo J, Li Y. Restoring Wnt/β-catenin signaling is a promising therapeutic strategy for Alzheimer’s disease. Mol Brain. 2019;12(1):104. 31801553 10.1186/s13041-019-0525-5 PMC6894260

[42] Yang C-P, Li X, Wu Y, et al Comprehensive integrative analyses identify GLT8D1 and CSNK2B as schizophrenia risk genes. Nat Commun. 2018;9(1):838. 29483533 10.1038/s41467-018-03247-3 PMC5826945

[43] Li L, Ghorbani M, Weisz-Hubshman M, et al Lysine acetyltransferase 8 is involved in cerebral development and syndromic intellectual disability. J Clin Invest. 2020;130(3):1431–1445. 31794431 10.1172/JCI131145 PMC7269600

[44] Tissink E, de Lange SC, Savage JE, et al Genome-wide association study of cerebellar volume provides insights into heritable mechanisms underlying brain development and mental health. Commun Biol. 2022;5(1):710. 35842455 10.1038/s42003-022-03672-7 PMC9288439

[45] Davies G, Lam M, Harris SE, et al Study of 300,486 individuals identifies 148 independent genetic loci influencing general cognitive function. Nat Commun. 2018;9(1):2098. 29844566 10.1038/s41467-018-04362-x PMC5974083

[46] Hagenaars SP, Harris SE, Davies G, et al Shared genetic aetiology between cognitive functions and physical and mental health in UK Biobank (N=112 151) and 24 GWAS consortia. Mol Psychiatry. 2016;21(11):1624–1632. 26809841 10.1038/mp.2015.225 PMC5078856

[47] Wood AR, Esko T, Yang J, et al Defining the role of common variation in the genomic and biological architecture of adult human height. Nat Genet. 2014;46(11):1173–1186. 25282103 10.1038/ng.3097 PMC4250049

[48] Davies G, Marioni RE, Liewald DC, et al Genome-wide association study of cognitive functions and educational attainment in UK Biobank (N=112 151). Mol Psychiatry. 2016;21(6):758–767. 27046643 10.1038/mp.2016.45 PMC4879186

[49] Wilkinson T, Schnier C, Bush K, et al Identifying dementia outcomes in UK Biobank: A validation study of primary care, hospital admissions and mortality data. Eur J Epidemiol. 2019;34(6):557–565. 30806901 10.1007/s10654-019-00499-1 PMC6497624

[50] Fry A, Littlejohns TJ, Sudlow C, et al Comparison of sociodemographic and health-related characteristics of UK Biobank participants with those of the general population. Am J Epidemiol. 2017;186(9):1026–1034. 28641372 10.1093/aje/kwx246 PMC5860371

