# Supplementary material for: Lung development genes, adult lung function and cognitive traits
Source: Brain Commun. 2024 Nov 1;6(6):fcae380. doi: 10.1093/braincomms/fcae380 (PMC11562126; doi:10.1093/braincomms/fcae380)
Supplement: fcae380_Supplementary_Data [file fcae380_supplementary_data.docx]

**Online supplementary materials**

**Lung development genes, adult lung function and cognitive traits**

**Corresponding author:**

Dr Mohammad Talaei, Centre for Preventive Neurology, Wolfson Institute of Population Health, Queen Mary University of London, Charterhouse Square, London EC1M 6B, UK. E-mail: [m.talaei@qmul.ac.uk](mailto:m.talaei@qmul.ac.uk); Tel: +44(0)20 7882 2499

Contents

[Further details 3](#_Toc180435336)

[Sample size 3](#_Toc180435337)

[Statistical analysis 3](#_Toc180435338)

[*Data wrangling for cognitive function tests* 3](#_Toc180435339)

[*Proportionality assumption* 4](#_Toc180435340)

[*Assumptions for linear regression* 4](#_Toc180435341)

[Latent general factor (g-factor) 4](#_Toc180435342)

[Summary statistics 5](#_Toc180435343)

[Colocalisation 5](#_Toc180435344)

[*Modified priors* 5](#_Toc180435345)

[References 6](#_Toc180435346)

[Supplementary tables and figure 7](#_Toc180435347)

**Supplementary** [**Figure 1.** Correlation between cognitive test scores and g-factors at UK biobank baseline (A) and Instance 2 (B) assessments. 7](#_Toc180435348)

**Supplementary** [**Table 1.** Summary of UKB variables used in the analysis. 8](#_Toc180435349)

**Supplementary** [**Table 2.** List of 55 lung developmental genes. 10](#_Toc180435350)

**Supplementary** [**Table 3.** Details of sample sizes for cognitive traits before and after exclusions. 11](#_Toc180435351)

**Supplementary** [**Table 4.** The proportional variance explained and the loadings of the individual cognitive tests for G-factors 12](#_Toc180435352)

**Supplementary** [**Table 5:** Linear regression coefficients (95% confidence interval) for cognitive function test scores according to forced vital capacity at baseline, adjusted for potential confounders in separate models. 13](#_Toc180435353)

**Supplementary** [**Table 6:** Linear regression coefficients (95% confidence interval) for cognitive function test scores according to forced expiratory volume in one second to forced vital capacity ratio at baseline, adjusted for potential confounders in separate models. 15](#_Toc180435354)

**Supplementary** [**Table 7:** Hazard ratio (95% confidence interval) for incident dementia according to lung function measures at baseline, adjusted for potential confounders in separate models. 17](#_Toc180435355)

**Supplementary** [**Table 8.** Colocalisation results for lung function measures and cognitive traits using default prior probabilities. 19](#_Toc180435356)

**Supplementary** [**Table 9.** Characteristics of variants and their effects where the lung function measure colocalised with cognitive traits or different variants were associated with each trait. 20](#_Toc180435357)

**Supplementary** [**Table 10:** Function and expression of genes with evidence of shared pathways for lung function and cognitive traits. 21](#_Toc180435358)

# Further details

## Sample size

The sample sizes varied according to the phenotypes analysed. Of 502,411 UK Biobank participants, the original sample size was 353,285 for lung function measures, ranging from 497,838 for the pairs matching test at baseline to 36,752 for the trail making test at instance 2, plus 8,022 cases of all-cause dementia. Supplementary Table 3 shows sample sizes after each exclusion. Exclusion criteria were as follows:

- non-Whites: based on self-reported ethnic background (UKB data field [21000](https://biobank.ndph.ox.ac.uk/showcase/field.cgi?id=21000)), including mixed, Asian or Asian British, Black or Black British, Chinese, other ethnic group, do not know, and Prefer not to answer.
- subjects with kinship coefficient ≥0.125; keeping one for each pair.
- subjects with poor heterozygosity/missingness: based on ‘het.missing.outliers’ (explained in [Resource 531](https://biobank.ndph.ox.ac.uk/ukb/refer.cgi?id=531)) which indicates samples identified as outliers in heterozygosity and missing rates (poor-quality genotypes).
- subjects without principal components (no genetic data).
- subjects with prevalent dementia at the time of cognitive function assessments (baseline or instance 2).

In summary, we excluded participants with a self-reported ethnicity other than White, who were a relative to another participant, had a poor-quality genotype (outliers in heterozygosity and missing rates), with no genetic data, or were already diagnosed with dementia at the time of cognitive function assessments. The actual sample sizes for genetic analysis after these exclusions were 306,476 for lung function measures, ranging from 428,609 to 32,321 for cognitive function tests, and 6,805 cases of all-cause dementia (421,241 controls), including 2,859 and 1,544 cases of Alzheimer’s disease and vascular dementia, respectively. Of these participants, those with lung function measures (used in observational analyses) ranged from 322,887 to 25,845 (depending on various cognitive function tests); we further excluded 120 cases with dementia at baseline resulting in 4,337 cases of incident all-cause dementia, including 1,882 and 958 cases of incident Alzheimer’s disease and vascular dementia, respectively.

## Statistical analysis

### *Data wrangling for cognitive function tests*

For the Symbol Digit test, we excluded cases with an extremely high number of attempts (>36). For the Matrix Pattern test, we used the ratio of the number of puzzles correctly solved (0-15) over the number of views (ranging 1-15), resulting in an index that ranges from 0 to 1. To measure executive function using the Trail Making test (TMT), we subtract the alphanumeric (TMT-B) from the numeric (TMT-A) path, resulting in TMT B-A.

### *Proportionality assumption*

We checked the proportionality assumption for Cox regression using the Stata *phtest* test based on Schoenfeld residuals after fitting models and visually using a log-log plot of survival (*stphplot*). We found no evidence of a violation of the proportionality assumption for the association of FVC with all-cause dementia (P=0.63), vascular dementia (P=0.75), and Alzheimer’s disease (P=0.19); also for the association of FEV_1_/FVC and Alzheimer’s disease (P=0.34). However, there were some indications for violation for the association of FEV_1_/FVC and all-cause dementia (P=0.03) and vascular dementia (P=0.02). However, further inspection of the log-log plots showed the violations were only in the middle quintiles and proportionality was met between extreme quintiles, particularly the top and the bottom quintiles. Moreover, logistic regression analysis, which has much more relaxed assumptions, resulted in odds ratios and 95% CIs that were very close to those obtained by Cox regression analysis for the associations between FEV_1_/FVC and all-cause dementia and vascular dementia.

### *Assumptions for linear regression*

We checked the normality of residuals visually using kernel density (*kdensity*), Q-Q (*qnorm*), and P-P (standardised normal probability; *pnorm*) plots. For the majority of nine cognitive function tests, particularly three g-factors, either residual had an approximately normal distribution, or there were only minor trivial deviations from normality. We also plotted the residuals versus fitted (predicted) values to explore the homogeneity of variance of the residuals (homoscedasticity). In most cases, there was no heteroscedasticity indication or only minor deviations. However, there were clear violations of these two assumptions for Trail Making B-A, as well as violations of normally distributed residual for Paired Learning, both consistently with FVC and FEV_1_/FVC. We also checked multicollinearity in all our models using the variance inflation factor (VIF). The largest VIF was only 5.23, and no value was above ten. Accordingly, the regression diagnostics we explored did not reveal major problems, although results from the two exceptions explained above should be interpreted with caution. However, our hypothesis did not rely on any single cognitive test, and the overall interpretation still remains valid.

## Latent general factor (g-factor)

We used confirmatory factor analysis (CFA) to estimate three latent variables with good model fit, Comparative Fit Index (CFI) and Tucker-Lewis Index (TLI) >0.92, and Root Mean Square Error of Approximation (RMSEA) and Standardized Root Mean Square Residual (SRMR) <0.06:

1. Using 4 cognitive tests at baseline: Pairs matching, Reaction Time, Fluid Intelligence/Reasoning, and Numeric Memory (CFI = 0.976, TLI = 0.927, RMSEA = 0.054, and SRMR = 0.022).
2. Using 5 tests at instance 2, termed “Executive Function” (1): Pairs matching, Reaction Time, Trail Making Test (alpha numeric minus numeric), [Symbol Digit Substitution](https://biobank.ndph.ox.ac.uk/ukb/field.cgi?id=23324), and [Tower Rearranging](https://biobank.ndph.ox.ac.uk/ukb/field.cgi?id=21004) (CFI = 0.995, TLI = 0.990, RMSEA = 0.022, and SRMR = 0.010).
3. Using all 9 tests at instance 2: CFI = 0.953, TLI = 0.938, RMSEA = 0.048, and SRMR = 0.028.

## Summary statistics

Genotyping and quality control of UK Biobank were explained in details elsewhere (<https://biobank.ctsu.ox.ac.uk/crystal/crystal/docs/genotyping_qc.pdf>).We obtained summary statistics (beta and standard error) for the association of eligible variants with phenotypes of interest using the Regenie program (2). We followed recommendations for UK Biobank analysis from pre-processing to step 2 (<https://rgcgithub.github.io/regenie/recommendations/>). Specifically, in step 1 of Regenie, we used a subset of SNPs with minor allele frequency ≥ 5%, genotyping rate ≥ 98.5%, Hardy-Weinberg Equilibrium test ≥ 1e-6, and minimum allele count at least 10 to calculate a leave-one-chromosome-out (LOCO) polygenic score for each trait and individual using Ridge regression adjusting for age, sex, genotyping array, assessment centre, height (only for FVC) and the top 10 ancestry principal components. Association testing was then performed, in step 2 of Regenie, including the LOCO polygenic predicted value from step 1 as an offset in the linear regression model, in addition to aforesaid same covariates. We used the approximate Firth likelihood ratio test for dementia as an unbalanced binary trait (Firth correction 0.01). Step 2 association tests were performed in a subset of 15,298 variants within previously reported 55 lung development genes (3).

## Colocalisation

For binary outcomes in colocalisation analyses, S (coloc command parameter indicating the proportion of samples that are cases) was set as 0.0159 for ACD, 0.0067 for AD, and 0.0037 for VD in UKB, and 0.1410 for AD in meta-GWAS data.

### *Modified priors*

To define modified prior probabilities taking into account prior information on the presence of at least one causal SNP for trait 1 in each gene, we used a *p1* (referring to trait 1 = lung function parameter) of 0.85 divided by number of SPNs. We derived *p2* and *p1|2* in a way to give the following probabilities (approximately):

o H0 of 0

o H1 of 0.85

o H2 of 0.05 (slightly more - 0.053)

o H3 of 0.05 (slightly less - 0.045)

o H4 of 0.05 (slightly more - 0.053)

This setting can be interpreted as an *a priori* hypothesis that 85% of our 55 genes are only associated with the lung function trait (H1), while we would expect 15% of them (8 genes) are associated with either only the cognitive trait (H2), both traits at different variants (H3), or both traits at the same variant (H4) – each with a probability of 5%. The probability of no association with either trait (H0) is 0. We used Stata codes below to define the SNP-level *p1*, *p2* and *p1|2* probabilities in order to get to a sum of the gene-level probabilities of H0 to H4 of 1 (as above):

*Stata codes* (Only input required being the number of SNPs)

** Overall (gene-level) prior probabilities

gen p1_g = 0.85

gen p2_g = (1-p1_g)/(2+p1_g)

gen p12_g = p2_g

gen sum_p_g = (p1_g + 2*p2_g + p12_g)

list n_snps p1_g p2_g p12_g sum_p_g

** SNP-level prior probabilities

gen p1 = p1_g/n_snps

gen p2 = p2_g/n_snps

gen p12 = p12_g/n_snps

gen sum_p = (p1 + 2*p2 + p12)*n_snps

list n_snps p1 p2 p12 sum_p

The output was verified by Prior explorer for coloc (<https://chr1swallace.shinyapps.io/coloc-priors/>) for random genes. The findings using these modified priors were presented in Supplementary Table 8.

# References

1. Tai XY, Chen C, Manohar S, et al. Impact of sleep duration on executive function and brain structure. *Commun Biol*. 2022;5(1):201.

2. Mbatchou J, Barnard L, Backman J, et al. Computationally efficient whole-genome regression for quantitative and binary traits. *Nat Genet*. 2021;53(7):1097-103.

3. Portas L, Pereira M, Shaheen SO, et al. Lung Development Genes and Adult Lung Function. *Am J Respir Crit Care Med*. 2020.

# Supplementary tables and figure

**A**


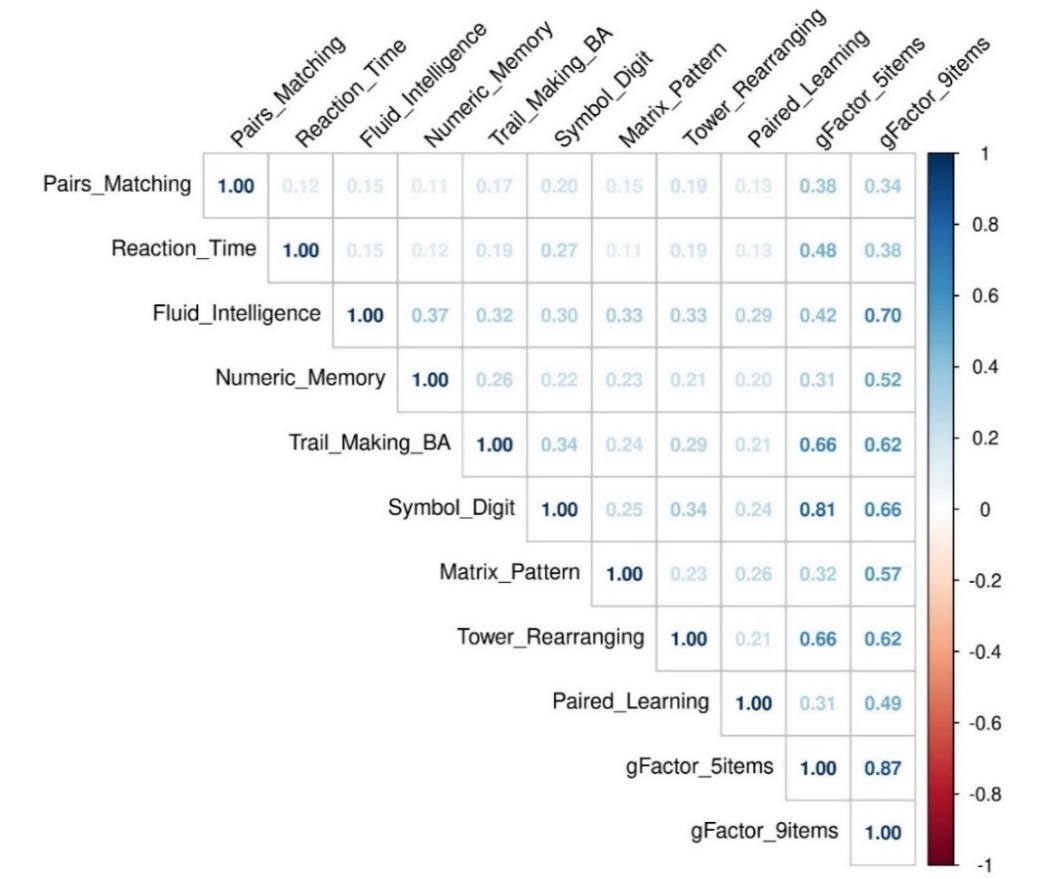


**B**

**
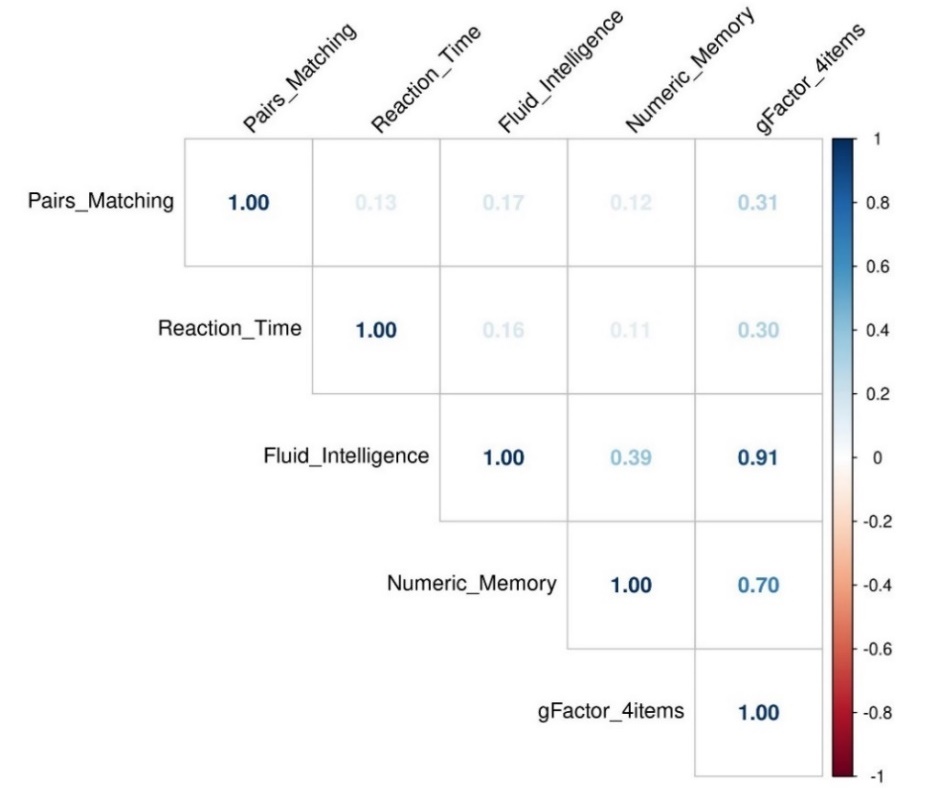
**

**Supplementary Figure 1.** Correlation between cognitive test scores and g-factors at UK biobank baseline (A) and Instance 2 (B) assessments.

Pearson correlation; N= 428,609 (Pairs Matching), 426,102 (Reaction Time), 138,885 (Fluid Intelligence), 43,508 (Numeric Memory), 32,321 (Trail Making B-A), 33,192 (Symbol Digit Substitution), 33,213 (Matrix Pattern Completion), 32,924 (Tower Rearranging), 33,583 (Paired Associate Learning), 42,602 (G-factor- 4 components at baseline), 44,643 (G-factor- 5 components at instance 2), 44,643 (G-factor- 9 components at instance 2).

**Supplementary Table 1.** Summary of UKB variables used in the analysis.

|  | **Field code** | **Time** | **Additional notes** |
| --- | --- | --- | --- |
| **Cognitive function tests** |  |  |  |
| [Pairs Matching](https://biobank.ndph.ox.ac.uk/ukb/field.cgi?id=399) | 399 | Baseline | Number of incorrect matches in round.0.1 |
| [Reaction Time](https://biobank.ndph.ox.ac.uk/ukb/field.cgi?id=20023) | 20023 | Baseline | Mean time to correctly identify matches.0.0 |
| [Fluid Intelligence](https://biobank.ndph.ox.ac.uk/ukb/field.cgi?id=20016) | 20016 | Baseline | Fluid intelligence score.0.0 |
| [Numeric Memory](https://biobank.ndph.ox.ac.uk/ukb/field.cgi?id=4282) | 4282 | Baseline | Maximum digits remembered correctly.0.0 |
| Trail Making |  |  |  |
| [Numeric](https://biobank.ndph.ox.ac.uk/ukb/field.cgi?id=6348) | 6348 | Instance 2 | Duration to complete numeric path (trail #1) .2.0 |
| [Alphanumeric](https://biobank.ndph.ox.ac.uk/ukb/field.cgi?id=6350) | 6350 | Instance 2 | Duration to complete alphanumeric path (trail #2).2.0 |
| [Symbol digit substitution](https://biobank.ndph.ox.ac.uk/ukb/field.cgi?id=23324) | 23324 | Instance 2 | Number of symbol digit matches made correctly.2.0 |
| Matrix Pattern Completion |  |  |  |
| [Score](https://biobank.ndph.ox.ac.uk/ukb/field.cgi?id=6373) | 6373 | Instance 2 | Number of puzzles correctly solved.2.0 |
| [Number of attempts](https://biobank.ndph.ox.ac.uk/ukb/field.cgi?id=6374) | 6374 | Instance 2 | Number of puzzles viewed.2.0 |
| [Tower Rearranging](https://biobank.ndph.ox.ac.uk/ukb/field.cgi?id=21004) | 21004 | Instance 2 | Number of puzzles correct.2.0 |
| [Paired Associate Learning](https://biobank.ndph.ox.ac.uk/ukb/field.cgi?id=20197) | 20197 | Instance 2 | Number of word pairs correctly associated.2.0 |
| **Dementia** |  |  |  |
| [All cause dementia](https://biobank.ndph.ox.ac.uk/ukb/field.cgi?id=41270)^*^ |  |  |  |
| Algorithmically defined | [42018](https://biobank.ctsu.ox.ac.uk/crystal/field.cgi?id=42018) | NA | Date of all cause dementia report |
|  | [42024](https://biobank.ctsu.ox.ac.uk/crystal/field.cgi?id=42024) | NA | Date of frontotemporal dementia report |
|  | [42020](https://biobank.ctsu.ox.ac.uk/crystal/field.cgi?id=42020) | NA | Date of Alzheimer’s disease report |
|  | [42022](https://biobank.ctsu.ox.ac.uk/crystal/field.cgi?id=42022) | NA | Date of vascular dementia report |
| First occurrences | [130836](https://biobank.ctsu.ox.ac.uk/crystal/field.cgi?id=130836) | NA | Date F00 first reported (dementia in Alzheimer’s disease) |
|  | [130838](https://biobank.ctsu.ox.ac.uk/crystal/field.cgi?id=130838) | NA | Date F01 first reported (vascular dementia) |
|  | [130840](https://biobank.ctsu.ox.ac.uk/crystal/field.cgi?id=130840) | NA | Date F02 first reported (dementia in other diseases classified elsewhere) |
|  | [130842](https://biobank.ctsu.ox.ac.uk/crystal/field.cgi?id=130842) | NA | Date F03 first reported (unspecified dementia) |
|  | [131036](https://biobank.ctsu.ox.ac.uk/crystal/field.cgi?id=131036) | NA | Date G30 first reported (Alzheimer’s disease) |
| [ICD10](https://biobank.ndph.ox.ac.uk/ukb/field.cgi?id=41270) | 41270 | NA | Diagnoses - ICD10: F00, F01, F02, F03, G30, G31, A81, F05.1, I67.3 |
| [Self-report](https://biobank.ctsu.ox.ac.uk/crystal/field.cgi?id=20002) | 20002 | NA | Non-cancer illness code, self-reported: dementia/Alzheimer’s/cognitive impairment |
| Alzheimer's disease^*^ |  |  |  |
| [Algorithmically defined](https://biobank.ctsu.ox.ac.uk/crystal/field.cgi?id=42020) | 42020 | NA | Date of Alzheimer’s disease report |
| First occurrences | [130836](https://biobank.ctsu.ox.ac.uk/crystal/field.cgi?id=130836) | NA | Date F00 first reported (dementia in Alzheimer’s disease) |
|  | [131036](https://biobank.ctsu.ox.ac.uk/crystal/field.cgi?id=131036) | NA | Date G30 first reported (Alzheimer’s disease) |
| [ICD10](https://biobank.ndph.ox.ac.uk/ukb/field.cgi?id=41270) | 41270 | NA | Diagnoses - ICD10: F00, G30 |
| Vascular dementia^*^ |  |  |  |
| [Algorithmically defined](https://biobank.ctsu.ox.ac.uk/crystal/field.cgi?id=42022) | 42022 | NA | Date of vascular dementia report |
| [First occurrences](https://biobank.ctsu.ox.ac.uk/crystal/field.cgi?id=130838) | 130838 | NA | Date F01 first reported (vascular dementia) |
| [ICD10](https://biobank.ndph.ox.ac.uk/ukb/field.cgi?id=41270) | 41270 | NA | Diagnoses - ICD10: F01 |
| **Lung function measures** |  |  |  |
| [FEV_1_ Best measure](https://biobank.ndph.ox.ac.uk/ukb/field.cgi?id=20150)^†^ | 20150 | Baseline | Forced expiratory volume in 1-second, Best measure.0.0 |
| [FVC Best measure](https://biobank.ndph.ox.ac.uk/ukb/field.cgi?id=20151)^†^ | 20151 | Baseline | Forced vital capacity (FVC), Best measure.0.0 |
| **Covariates** |  |  |  |
| [Age](https://biobank.ndph.ox.ac.uk/ukb/field.cgi?id=21003) | 21003 | Baseline | Age when attended assessment centre.0.0 |
| [Sex](https://biobank.ndph.ox.ac.uk/ukb/field.cgi?id=31) | 31 | Baseline | Sex.0.0 |
| [Height](https://biobank.ndph.ox.ac.uk/showcase/field.cgi?id=50) | 50 | Baseline | Standing height.0.0 |
| [Ethnicity](https://biobank.ndph.ox.ac.uk/showcase/field.cgi?id=21000) | 21000 | Baseline | Ethnic background.0.0 |
| [Townsend deprivation index](https://biobank.ndph.ox.ac.uk/ukb/field.cgi?id=189) | 189 | Baseline | Townsend deprivation index at recruitment.0.0 |
| [Education](https://biobank.ndph.ox.ac.uk/ukb/field.cgi?id=6138) | 6138 | Baseline | Qualifications.0.0 |
| [Smoking](https://biobank.ndph.ox.ac.uk/showcase/field.cgi?id=20116) | 20166 | Baseline | Smoking status.0.0 |
| [Centre](https://biobank.ndph.ox.ac.uk/ukb/field.cgi?id=54) | 54 | Baseline | UK Biobank assessment centre.0.0 |
| [Batch](https://biobank.ndph.ox.ac.uk/ukb/field.cgi?id=22000) | 22000 | Baseline | Genotype measurement batch.0.0 |
| [PC 1-10](https://biobank.ndph.ox.ac.uk/ukb/field.cgi?id=22009) | 22009 | Baseline | Genetic principal components.0.1-.0.10 |
| [Date lost to follow-up](https://biobank.ndph.ox.ac.uk/showcase/field.cgi?tk=fY4kA0K2LZWXc8Yii37OzwePk1OqXgG8421957&id=191) | 191 | NA | - |

^*^ We updated Algorithmically defined variables using the First Occurrences data that reported dementia onset (within nervous system disorders and mental and behavioural disorders).

Algorithmically defined outcomes (UKB Resource 594) include UKB self-report, ICD-9, ICD-10,

and Read codes. Further details were provided here: <https://community.ukbiobank.ac.uk/hc/en-gb/articles/15467565352093-Health-related-outcomes-data>.

^†^ Highest measure from the array of values for Forced Expiratory Volume in 1-second (FEV1) [Field 3063](https://biobank.ndph.ox.ac.uk/ukb/field.cgi?id=3063), or highest measure from the array of values for Forced Vital Capacity (FVC) ([Field 3062](https://biobank.ndph.ox.ac.uk/ukb/field.cgi?id=3062)), which were "acceptable" according to corresponding acceptability field ([Field 3061](https://biobank.ndph.ox.ac.uk/ukb/field.cgi?id=3061)).

**Supplementary Table 2.** List of 55 lung developmental genes.

| **Gene** | **Gene** |
| --- | --- |
| **FVC** | **FEV_1_/FVC** |
| *AGER* | *AGER* |
| *HHIP* | *HHIP* |
| *PTCH1* | *PTCH1* |
| *SOX9* | *SOX9* |
| *TNS1* | *TNS1* |
| *ACTN3* | *FGF18* |
| *ACTN4* | *FGFR3* |
| *CLDN20* | *FGFR4* |
| *FGF10* | *GATA6* |
| *GSK3B* | *GFI1* |
| *HOXA1* | *GJE1* |
| *HOXB4* | *ITGAV* |
| *IGF1* | *KAT7* |
| *ITGB5* | *MAPRE1* |
| *KAT8* | *MMP15* |
| *KDR* | *NFATC3* |
| *MMP24* | *PARD3* |
| *NCOR1* | *PDGFB* |
| *NCOR2* | *PPARD* |
| *NR3C1* | *RARA* |
| *RARB* | *RUNX2* |
| *ROR2* | *RUNX3* |
| *RUNX1* | *SERPING1* |
| *SERPINC1* | *SFRP2* |
| *WNT2B* | *SFTPD* |
| *WNT9A* | *TCF7L1* |
|  | *TGFB2* |
|  | *WNT7A* |
|  | *BMP4* |
|  | *CSNK2B* |
|  | *CTNND1* |
|  | *DSP* |
|  | *ELN* |
|  | *FARP2* |

Derived from Portas et al. DOI: 10.1164/rccm.201912-2338OC.

**Supplementary Table 3.** Details of sample sizes for cognitive traits before and after exclusions.

|  | **Original** |  | **Exclusion criteria** | | |  |  |  |
| --- | --- | --- | --- | --- | --- | --- | --- | --- |
|  |  | **Ethnic^*^** | **Kinship^†^** | **Outlier^‡^** | **PC^§^** | **Dementia^#^** | **LF**^¶^ | **Covar^⁑^** |
| **Cognitive function tests** |  |  |  |  |  |  |  |  |
| Pairs Matching | 497,838 | 469,023 | 439,005 | 438,100 | 428,700 | 428,609 | 322,887 | 321,407 |
| [Reaction Time](https://biobank.ndph.ox.ac.uk/ukb/field.cgi?id=20023) | 496,581 | 469,312 | 439,453 | 438,552 | 426,181 | 426,102 | 321,151 | 319,700 |
| [Fluid Intelligence](https://biobank.ndph.ox.ac.uk/ukb/field.cgi?id=20016) | 165,427 | 152,510 | 143,658 | 143,359 | 138,915 | 138,885 | 105,592 | 105,121 |
| [Numeric Memory](https://biobank.ndph.ox.ac.uk/ukb/field.cgi?id=4282) | 50,353 | 47,823 | 44,467 | 44,384 | 43,516 | 43,508 | 33,902 | 33,722 |
| Trail Making B-A^**^ | 36,752 | 35,605 | 33,307 | 33,248 | 32,334 | 32,321 | 25,845 | 25,777 |
| [Symbol Digit Substitution](https://biobank.ndph.ox.ac.uk/ukb/field.cgi?id=23324) | 37,748 | 36,556 | 34,195 | 34,134 | 33,206 | 33,192 | 26,525 | 26,454 |
| [Matrix Pattern Completion](https://biobank.ndph.ox.ac.uk/ukb/field.cgi?id=6373) | 37,755 | 36,573 | 34,214 | 34,153 | 33,226 | 33,213 | 26,545 | 26,474 |
| [Tower Rearranging](https://biobank.ndph.ox.ac.uk/ukb/field.cgi?id=21004) | 37,416 | 36,252 | 33,915 | 33,854 | 32,936 | 32,924 | 26,324 | 26,256 |
| [Paired Associate Learning](https://biobank.ndph.ox.ac.uk/ukb/field.cgi?id=20197) | 38,207 | 36,995 | 34,604 | 34,543 | 33,597 | 33,583 | 26,815 | 26,743 |
| **G-factor**^††^ |  |  |  |  |  |  |  |  |
| 4 components at baseline^‡‡^ | 48,741 | 46,809 | 43,529 | 43,448 | 42,610 | 42,602 | 33,310 | 33,142 |
| 5 components at instance 2^§§^ | 50,746 | 49,125 | 45,960 | 45,880 | 44,661 | 44,643 | 35,593 | 35,497 |
| 9 components at instance 2 | 50,746 | 49,125 | 45,960 | 45,880 | 44,661 | 44,643 | 35,593 | 35,497 |
| [**All Cause Dementia**](https://biobank.ndph.ox.ac.uk/ukb/field.cgi?id=41270) |  |  |  |  |  |  |  |  |
| Cases | 8,022 | 7,631 | 7,121 | 7,102 | 6,805 | 6,805 | 4,457 | 4,294 |
| Non-cases | 494,389 | 464,980 | 435,442 | 434,554 | 422,292 | 421,241 | 317,660 | 316,229 |
| Total UKB participants | 502,411 | 472,611 | 442,563 | 441,656 | 429,097 | 428,046 | 322,117 | 320,523 |

PC: Principal Components, UKB: UK Biobank, LF: Lung function measure, Covar: Covariate. G-factor indicates general cognitive ability.

^*^ non-Whites.

^†^ Relatives (kinship coefficient ≥0.125; keeping one for each pair).

^‡^ Poor heterozygosity/missingness.

^§^ Participants without Principal Components (genetic data).

^#^ Excluding prevalent dementia at the time of CF measurements; *this column shows sample sizes used for genetic analyses***.**

^¶^ Participants without data on lung function were excluded from observational analysis.

^⁑^ Participants without data on covariates were excluded; *this column shows sample sizes used for observational analyses***.**

^**^ Alphanumeric (B) – Numeric (A).

^††^ Measures of general cognitive ability (g-factor) using confirmatory factor analysis.

^‡‡^ Pairs Matching, Reaction Time, Fluid Intelligence, and Numeric Memory.

^§§^ Measure of working memory and speed of processing termed “Executive Function” including Pairs Matching, Reaction Time, Trail Making B-A, Digit Symbol, and Tower Rearranging.

**Supplementary** Table 4. The proportional variance explained and the loadings of the individual cognitive tests for G-factors

|  | **Baseline** | | **Visit 2** | | | |
| --- | --- | --- | --- | --- | --- | --- |
|  | **Four tests** | | **Executive Function** | | **All tests** | |
|  | **Loading** | **Variance** | **Loading** | **Variance** | **Loading** | **Variance** |
| Pairs matching | 0.236 | 0.056 | 0.314 | 0.099 | 0.291 | 0.085 |
| Reaction time | 0.247 | 0.061 | 0.406 | 0.165 | 0.348 | 0.121 |
| Prospective memory |  |  |  |  |  |  |
| Fluid intelligence | 0.710 | 0.504 |  |  | 0.631 | 0.398 |
| Numeric memory | 0.548 | 0.300 |  |  | 0.477 | 0.228 |
| Trail making |  |  | 0.564 | 0.318 | 0.580 | 0.336 |
| Symbol digit substitution |  |  | 0.672 | 0.452 | 0.601 | 0.362 |
| Matrix pattern completion |  |  | 0.552 | 0.304 | 0.632 | 0.399 |
| Tower rearranging |  |  |  |  | 0.569 | 0.324 |
| Paired associate learning |  |  |  |  | 0.462 | 0.213 |

**Supplementary** Table 5: Linear regression coefficients (95% confidence interval) for cognitive function test scores according to forced vital capacity at baseline, adjusted for potential confounders in separate models.

|  |  | **Quintiles of FVC** | | |  |  | **Per SD** |
| --- | --- | --- | --- | --- | --- | --- | --- |
|  | **Q1** | **Q2** | **Q3** | **Q4** | **Q5** | **P-trend** |  |
| **Median (IQR), L** | 2.62 (2.37-2.78) | 3.17 (3.05-3.29) | 3.65 (3.53-3.78) | 4.24 (4.08-4.42) | 5.12 (4.84-5.53) |  |  |
| **Pairs Matching** |  |  |  |  |  |  |  |
| Model 1 | 0.00 | 0.08 (0.07, 0.09) | 0.11 (0.10, 0.12) | 0.13 (0.12, 0.14) | 0.19 (0.18, 0.20) | <0.001 | 0.06 (0.06, 0.07) |
| Model 2 | 0.00 | 0.01 (0.00, 0.02) | 0.00 (-0.01, 0.02) | 0.00 (-0.01, 0.02) | 0.01 (-0.01, 0.02) | 0.94 | -0.00 (-0.01, 0.00) |
| Model 3 | 0.00 | 0.02 (0.00, 0.03) | 0.01 (-0.00, 0.02) | 0.01 (-0.00, 0.02) | 0.02 (0.00, 0.03) | 0.12 | 0.00 (-0.00, 0.01) |
| **Reaction Time** |  |  |  |  |  |  |  |
| Model 1 | 0.00 | 0.19 (0.18, 0.20) | 0.31 (0.30, 0.32) | 0.39 (0.38, 0.40) | 0.56 (0.55, 0.57) | <0.001 | 0.19 (0.19, 0.20) |
| Model 2 | 0.00 | 0.06 (0.05, 0.07) | 0.10 (0.08, 0.11) | 0.13 (0.12, 0.14) | 0.17 (0.16, 0.19) | <0.001 | 0.06 (0.06, 0.07) |
| Model 3 | 0.00 | 0.04 (0.03, 0.05) | 0.07 (0.06, 0.08) | 0.10 (0.09, 0.12) | 0.14 (0.12, 0.15) | <0.001 | 0.05 (0.05, 0.06) |
| **Fluid Intelligence** |  |  |  |  |  |  |  |
| Model 1 | 0.00 | 0.16 (0.14, 0.18) | 0.23 (0.22, 0.25) | 0.30 (0.28, 0.32) | 0.41 (0.39, 0.43) | <0.001 | 0.14 (0.13, 0.15) |
| Model 2 | 0.00 | 0.10 (0.08, 0.12) | 0.15 (0.12, 0.17) | 0.20 (0.18, 0.22) | 0.25 (0.23, 0.28) | <0.001 | 0.09 (0.08, 0.10) |
| Model 3 | 0.00 | 0.04 (0.02, 0.05) | 0.06 (0.04, 0.08) | 0.09 (0.07, 0.11) | 0.13 (0.10, 0.15) | <0.001 | 0.05 (0.04, 0.06) |
| **Numeric Memory** |  |  |  |  |  |  |  |
| Model 1 | 0.00 | 0.17 (0.13, 0.20) | 0.26 (0.23, 0.29) | 0.31 (0.28, 0.35) | 0.46 (0.43, 0.49) | <0.001 | 0.16 (0.15, 0.17) |
| Model 2 | 0.00 | 0.11 (0.07, 0.14) | 0.17 (0.13, 0.20) | 0.20 (0.16, 0.24) | 0.29 (0.24, 0.34) | <0.001 | 0.11 (0.09, 0.13) |
| Model 3 | 0.00 | 0.06 (0.03, 0.09) | 0.11 (0.07, 0.14) | 0.13 (0.09, 0.17) | 0.20 (0.15, 0.25) | <0.001 | 0.08 (0.06, 0.10) |
| **Trail Making B-A** |  |  |  |  |  |  |  |
| Model 1 | 0.00 | 0.17 (0.13, 0.22) | 0.25 (0.21, 0.30) | 0.25 (0.21, 0.29) | 0.33 (0.29, 0.37) | <0.001 | 0.09 (0.08, 0.11) |
| Model 2 | 0.00 | 0.07 (0.02, 0.11) | 0.10 (0.06, 0.15) | 0.13 (0.08, 0.18) | 0.16 (0.10, 0.22) | <0.001 | 0.04 (0.02, 0.06) |
| Model 3 | 0.00 | 0.04 (0.00, 0.09) | 0.08 (0.03, 0.12) | 0.10 (0.05, 0.15) | 0.12 (0.06, 0.18) | <0.001 | 0.03 (0.01, 0.05) |
| **Symbol Digit** |  |  |  |  |  |  |  |
| Model 1 | 0.00 | 0.21 (0.17, 0.26) | 0.30 (0.26, 0.34) | 0.30 (0.26, 0.34) | 0.42 (0.38, 0.46) | <0.001 | 0.13 (0.12, 0.14) |
| Model 2 | 0.00 | 0.04 (-0.00, 0.08) | 0.06 (0.02, 0.10) | 0.10 (0.06, 0.15) | 0.14 (0.09, 0.20) | <0.001 | 0.06 (0.04, 0.07) |
| Model 3 | 0.00 | 0.01 (-0.03, 0.05) | 0.02 (-0.02, 0.06) | 0.05 (0.00, 0.10) | 0.08 (0.02, 0.13) | 0.002 | 0.04 (0.02, 0.05) |
| **Matrix Pattern** |  |  |  |  |  |  |  |
| Model 1 | 0.00 | 0.17 (0.13, 0.22) | 0.21 (0.17, 0.26) | 0.31 (0.27, 0.35) | 0.43 (0.39, 0.47) | <0.001 | 0.14 (0.13, 0.15) |
| Model 2 | 0.00 | 0.09 (0.05, 0.14) | 0.09 (0.04, 0.13) | 0.15 (0.10, 0.21) | 0.21 (0.15, 0.27) | <0.001 | 0.07 (0.05, 0.09) |
| Model 3 | 0.00 | 0.05 (0.01, 0.10) | 0.03 (-0.01, 0.08) | 0.08 (0.03, 0.13) | 0.13 (0.07, 0.19) | <0.001 | 0.05 (0.03, 0.06) |
| **Tower Rearranging** |  |  |  |  |  |  |  |
| Model 1 | 0.00 | 0.13 (0.09, 0.18) | 0.23 (0.19, 0.28) | 0.31 (0.26, 0.35) | 0.41 (0.36, 0.45) | <0.001 | 0.13 (0.12, 0.15) |
| Model 2 | 0.00 | 0.02 (-0.02, 0.07) | 0.04 (-0.00, 0.09) | 0.06 (0.01, 0.12) | 0.06 (-0.00, 0.12) | 0.07 | 0.02 (-0.00, 0.03) |
| Model 3 | 0.00 | 0.00 (-0.04, 0.05) | 0.02 (-0.02, 0.07) | 0.04 (-0.01, 0.09) | 0.04 (-0.02, 0.09) | 0.20 | 0.01 (-0.01, 0.03) |
| **Paired Learning** |  |  |  |  |  |  |  |
| Model 1 | 0.00 | 0.14 (0.10, 0.18) | 0.13 (0.08, 0.17) | 0.04 (-0.00, 0.08) | 0.00 (-0.04, 0.04) | <0.001 | -0.03 (-0.04, -0.02) |
| Model 2 | 0.00 | 0.09 (0.04, 0.13) | 0.11 (0.06, 0.15) | 0.18 (0.13, 0.23) | 0.20 (0.14, 0.26) | <0.001 | 0.05 (0.03, 0.07) |
| Model 3 | 0.00 | 0.05 (0.01, 0.09) | 0.05 (0.01, 0.10) | 0.11 (0.06, 0.16) | 0.12 (0.07, 0.18) | <0.001 | 0.03 (0.01, 0.05) |
| **G-factors** |  |  |  |  |  |  |  |
| **4 components at baseline** |  |  |  |  |  |  |  |
| Model 1 | 0.00 | 0.19 (0.16, 0.21) | 0.25 (0.23, 0.28) | 0.31 (0.28, 0.33) | 0.44 (0.42, 0.47) | <0.001 | 0.15 (0.14, 0.15) |
| Model 2 | 0.00 | 0.11 (0.08, 0.14) | 0.13 (0.11, 0.16) | 0.16 (0.13, 0.20) | 0.23 (0.19, 0.26) | <0.001 | 0.08 (0.06, 0.09) |
| Model 3 | 0.00 | 0.06 (0.04, 0.08) | 0.07 (0.04, 0.09) | 0.09 (0.05, 0.12) | 0.13 (0.09, 0.16) | <0.001 | 0.05 (0.03, 0.06) |
| **5 components at Instance 2** |  |  |  |  |  |  |  |
| Model 1 | 0.00 | 0.16 (0.13, 0.18) | 0.25 (0.22, 0.28) | 0.29 (0.26, 0.32) | 0.39 (0.37, 0.42) | <0.001 | 0.13 (0.12, 0.14) |
| Model 2 | 0.00 | 0.03 (0.00, 0.06) | 0.06 (0.03, 0.08) | 0.10 (0.07, 0.13) | 0.11 (0.07, 0.14) | <0.001 | 0.04 (0.03, 0.05) |
| Model 3 | 0.00 | 0.01 (-0.01, 0.04) | 0.03 (0.00, 0.06) | 0.06 (0.03, 0.09) | 0.07 (0.04, 0.11) | <0.001 | 0.03 (0.02, 0.04) |
| **9 components at Instance 2** |  |  |  |  |  |  |  |
| Model 1 | 0.00 | 0.20 (0.17, 0.23) | 0.29 (0.26, 0.32) | 0.36 (0.33, 0.38) | 0.48 (0.45, 0.51) | <0.001 | 0.15 (0.14, 0.16) |
| Model 2 | 0.00 | 0.08 (0.05, 0.11) | 0.11 (0.08, 0.14) | 0.17 (0.14, 0.20) | 0.20 (0.16, 0.24) | <0.001 | 0.06 (0.05, 0.08) |
| Model 3 | 0.00 | 0.04 (0.01, 0.07) | 0.06 (0.03, 0.09) | 0.10 (0.07, 0.14) | 0.13 (0.09, 0.17) | <0.001 | 0.04 (0.03, 0.06) |

FVC: forced vital capacity. G-factor indicates general cognitive ability.

Model 1: crude.

Model 2: adjusted for age, sex, height, assessment centre, and Townsend deprivation index.

Model 3: further adjusted for 12 potentially modifiable risk factors for dementia including education, hearing loss, traumatic brain injury, hypertension, alcohol >21 units per week, obesity, smoking, depression, social isolation, physical inactivity, air pollution, and diabetes. The model presented in the main manuscript includes APOE4 alleles as well.

**Supplementary** Table 6: Linear regression coefficients (95% confidence interval) for cognitive function test scores according to forced expiratory volume in one second to forced vital capacity ratio at baseline, adjusted for potential confounders in separate models.

|  |  | **Quintiles of** **FEV_1_/FVC** | | |  |  | **Per SD** |
| --- | --- | --- | --- | --- | --- | --- | --- |
|  | **Q1** | **Q2** | **Q3** | **Q4** | **Q5** | **P-trend** |  |
| **Median (IQR)** | 0.68 (0.64-0.70) | 0.73 (0.72-0.74) | 0.76 (0.76-0.77) | 0.79 (0.78-0.80) | 0.82 (0.81-0.84) |  |  |
| **Pairs Matching** |  |  |  |  |  |  |  |
| Model 1 | 0.00 | 0.03 (0.02, 0.04) | 0.05 (0.04, 0.06) | 0.08 (0.07, 0.09) | 0.12 (0.11, 0.13) | <0.001 | 0.04 (0.04, 0.04) |
| Model 2 | 0.00 | 0.00 (-0.01, 0.01) | 0.01 (0.00, 0.02) | 0.02 (0.01, 0.03) | 0.02 (0.01, 0.04) | <0.001 | 0.01 (0.01, 0.01) |
| Model 3 | 0.00 | 0.00 (-0.01, 0.01) | 0.01 (0.00, 0.02) | 0.02 (0.01, 0.03) | 0.02 (0.01, 0.03) | <0.001 | 0.01 (0.00, 0.01) |
| **Reaction Time** |  |  |  |  |  |  |  |
| Model 1 | 0.00 | 0.08 (0.07, 0.09) | 0.10 (0.09, 0.12) | 0.14 (0.13, 0.15) | 0.22 (0.21, 0.23) | <0.001 | 0.08 (0.08, 0.08) |
| Model 2 | 0.00 | 0.03 (0.02, 0.04) | 0.03 (0.02, 0.04) | 0.03 (0.02, 0.04) | 0.03 (0.02, 0.04) | <0.001 | 0.02 (0.01, 0.02) |
| Model 3 | 0.00 | 0.02 (0.01, 0.03) | 0.02 (0.01, 0.03) | 0.02 (0.01, 0.03) | 0.02 (0.01, 0.03) | 0.004 | 0.01 (0.01, 0.02) |
| **Fluid Intelligence** |  |  |  |  |  |  |  |
| Model 1 | 0.00 | 0.04 (0.02, 0.06) | 0.05 (0.03, 0.07) | 0.06 (0.04, 0.07) | 0.09 (0.07, 0.10) | <0.001 | 0.03 (0.03, 0.04) |
| Model 2 | 0.00 | 0.03 (0.02, 0.05) | 0.04 (0.02, 0.05) | 0.03 (0.01, 0.05) | 0.04 (0.02, 0.06) | <0.001 | 0.02 (0.01, 0.02) |
| Model 3 | 0.00 | 0.01 (-0.01, 0.03) | 0.00 (-0.01, 0.02) | 0.00 (-0.02, 0.02) | 0.01 (-0.00, 0.03) | 0.31 | 0.00 (-0.00, 0.01) |
| **Numeric Memory** |  |  |  |  |  |  |  |
| Model 1 | 0.00 | 0.00 (-0.03, 0.04) | 0.03 (-0.01, 0.06) | 0.02 (-0.01, 0.05) | 0.06 (0.02, 0.09) | <0.001 | 0.02 (0.01, 0.03) |
| Model 2 | 0.00 | -0.01 (-0.04, 0.03) | 0.01 (-0.03, 0.04) | -0.01 (-0.04, 0.02) | -0.01 (-0.04, 0.03) | 0.71 | 0.00 (-0.01, 0.01) |
| Model 3 | 0.00 | -0.01 (-0.04, 0.02) | 0.01 (-0.03, 0.04) | -0.01 (-0.04, 0.03) | 0.01 (-0.03, 0.04) | 0.78 | 0.00 (-0.01, 0.01) |
| **Trail Making B-A** |  |  |  |  |  |  |  |
| Model 1 | 0.00 | 0.09 (0.05, 0.13) | 0.12 (0.08, 0.16) | 0.16 (0.12, 0.20) | 0.21 (0.17, 0.25) | <0.001 | 0.08 (0.07, 0.10) |
| Model 2 | 0.00 | 0.06 (0.02, 0.09) | 0.06 (0.02, 0.10) | 0.06 (0.03, 0.10) | 0.06 (0.02, 0.09) | 0.003 | 0.03 (0.01, 0.04) |
| Model 3 | 0.00 | 0.05 (0.01, 0.09) | 0.06 (0.02, 0.09) | 0.06 (0.02, 0.10) | 0.05 (0.01, 0.09) | 0.004 | 0.03 (0.01, 0.04) |
| **Symbol Digit** |  |  |  |  |  |  |  |
| Model 1 | 0.00 | 0.10 (0.06, 0.14) | 0.14 (0.10, 0.18) | 0.22 (0.18, 0.26) | 0.31 (0.27, 0.34) | <0.001 | 0.11 (0.10, 0.13) |
| Model 2 | 0.00 | 0.05 (0.01, 0.08) | 0.03 (-0.01, 0.06) | 0.04 (0.01, 0.08) | 0.03 (-0.00, 0.07) | 0.09 | 0.01 (0.00, 0.03) |
| Model 3 | 0.00 | 0.04 (0.01, 0.08) | 0.03 (-0.01, 0.06) | 0.05 (0.01, 0.08) | 0.04 (0.00, 0.07) | 0.04 | 0.02 (0.00, 0.03) |
| **Matrix Pattern** |  |  |  |  |  |  |  |
| Model 1 | 0.00 | 0.05 (0.01, 0.09) | 0.06 (0.02, 0.10) | 0.07 (0.03, 0.11) | 0.09 (0.05, 0.13) | <0.001 | 0.04 (0.02, 0.05) |
| Model 2 | 0.00 | 0.04 (0.00, 0.08) | 0.03 (-0.01, 0.07) | 0.03 (-0.01, 0.07) | 0.01 (-0.03, 0.05) | 0.73 | 0.00 (-0.01, 0.02) |
| Model 3 | 0.00 | 0.03 (-0.00, 0.07) | 0.03 (-0.01, 0.07) | 0.03 (-0.01, 0.07) | 0.01 (-0.03, 0.04) | 0.67 | 0.01 (-0.01, 0.02) |
| **Tower Rearranging** |  |  |  |  |  |  |  |
| Model 1 | 0.00 | 0.04 (-0.00, 0.08) | 0.04 (0.00, 0.08) | 0.12 (0.08, 0.16) | 0.16 (0.12, 0.20) | <0.001 | 0.07 (0.05, 0.08) |
| Model 2 | 0.00 | 0.02 (-0.01, 0.06) | 0.00 (-0.04, 0.04) | 0.06 (0.02, 0.10) | 0.04 (0.00, 0.08) | 0.01 | 0.02 (0.01, 0.04) |
| Model 3 | 0.00 | 0.02 (-0.02, 0.06) | 0.00 (-0.04, 0.04) | 0.06 (0.02, 0.10) | 0.04 (0.00, 0.08) | 0.008 | 0.02 (0.01, 0.04) |
| **Paired Learning** |  |  |  |  |  |  |  |
| Model 1 | 0.00 | 0.06 (0.02, 0.10) | 0.11 (0.07, 0.15) | 0.15 (0.11, 0.19) | 0.17 (0.13, 0.21) | <0.001 | 0.07 (0.05, 0.08) |
| Model 2 | 0.00 | 0.01 (-0.03, 0.05) | 0.03 (-0.01, 0.07) | 0.03 (-0.00, 0.07) | 0.02 (-0.02, 0.05) | 0.19 | 0.01 (-0.00, 0.02) |
| Model 3 | 0.00 | 0.01 (-0.03, 0.05) | 0.03 (-0.00, 0.07) | 0.05 (0.01, 0.08) | 0.03 (-0.01, 0.07) | 0.02 | 0.01 (0.00, 0.03) |
| **G-factors** |  |  |  |  |  |  |  |
| **4 components at baseline** |  |  |  |  |  |  |  |
| Model 1 | 0.00 | 0.01 (-0.01, 0.04) | 0.04 (0.01, 0.06) | 0.04 (0.01, 0.07) | 0.08 (0.05, 0.11) | <0.001 | 0.03 (0.02, 0.04) |
| Model 2 | 0.00 | 0.00 (-0.02, 0.03) | 0.01 (-0.01, 0.04) | 0.00 (-0.02, 0.03) | 0.01 (-0.01, 0.04) | 0.44 | 0.00 (-0.00, 0.01) |
| Model 3 | 0.00 | -0.01 (-0.03, 0.02) | 0.00 (-0.02, 0.02) | -0.01 (-0.03, 0.02) | 0.01 (-0.02, 0.03) | 0.72 | 0.00 (-0.01, 0.01) |
| **5 components at Instance 2** |  |  |  |  |  |  |  |
| Model 1 | 0.00 | 0.08 (0.05, 0.10) | 0.09 (0.07, 0.12) | 0.16 (0.13, 0.18) | 0.22 (0.20, 0.25) | <0.001 | 0.08 (0.08, 0.09) |
| Model 2 | 0.00 | 0.04 (0.02, 0.06) | 0.03 (0.01, 0.05) | 0.05 (0.03, 0.07) | 0.04 (0.02, 0.06) | 0.001 | 0.02 (0.01, 0.03) |
| Model 3 | 0.00 | 0.04 (0.02, 0.06) | 0.03 (0.01, 0.05) | 0.05 (0.03, 0.07) | 0.04 (0.02, 0.06) | <0.001 | 0.02 (0.01, 0.03) |
| **9 components at Instance 2** |  |  |  |  |  |  |  |
| Model 1 | 0.00 | 0.07 (0.04, 0.10) | 0.09 (0.06, 0.11) | 0.14 (0.11, 0.17) | 0.20 (0.17, 0.22) | <0.001 | 0.08 (0.07, 0.09) |
| Model 2 | 0.00 | 0.04 (0.01, 0.06) | 0.03 (0.00, 0.05) | 0.04 (0.02, 0.07) | 0.03 (0.00, 0.06) | 0.03 | 0.01 (0.01, 0.02) |
| Model 3 | 0.00 | 0.03 (0.01, 0.06) | 0.03 (0.01, 0.05) | 0.04 (0.02, 0.07) | 0.03 (0.01, 0.06) | 0.004 | 0.02 (0.01, 0.03) |

FVC: forced vital capacity; FEV_1_: forced expiratory volume in one second. G-factor indicates general cognitive ability.

Model 1: crude.

Model 2: adjusted for age, sex, assessment centre, Townsend deprivation index, and education.

Model 3: further adjusted for 12 potentially modifiable risk factors for dementia including education, hearing loss, traumatic brain injury, hypertension, alcohol >21 units per week, obesity, smoking, depression, social isolation, physical inactivity, air pollution, and diabetes. The model presented in the main manuscript includes APOE4 alleles as well.

**Supplementary** Table 7: Hazard ratio (95% confidence interval) for incident dementia according to lung function measures at baseline, adjusted for potential confounders in separate models.

|  |  | **Quintiles of lung function measures** | | |  |  | **Per SD** |
| --- | --- | --- | --- | --- | --- | --- | --- |
|  | **Q1** | **Q2** | **Q3** | **Q4** | **Q5** | **P-trend** |  |
| **FVC** |  |  |  |  |  |  |  |
| **All-cause dementia** |  |  |  |  |  |  |  |
| **Cases/person-years** | 1,415/829,954 | 868/864,434 | 803/869,355 | 790/874,851 | 461/888,413 |  |  |
| Model 1 | 1.00 | 0.58 (0.53-0.63) | 0.53 (0.49-0.58) | 0.52 (0.48-0.57) | 0.30 (0.27-0.33) | <0.001 | 0.65 (0.63-0.67) |
| Model 2 | 1.00 | 0.78 (0.71-0.85) | 0.69 (0.62-0.76) | 0.61 (0.54-0.68) | 0.49 (0.43-0.57) | <0.001 | 0.75 (0.71-0.79) |
| Model 3 | 1.00 | 0.83 (0.76-0.91) | 0.77 (0.70-0.86) | 0.72 (0.64-0.81) | 0.62 (0.54-0.72) | <0.001 | 0.82 (0.78-0.87) |
| **Vascular dementia** |  |  |  |  |  |  |  |
| **Cases/person-years** | 322/829,954 | 200/864,434 | 175/869,355 | 168/874,851 | 93/888,413 |  |  |
| Model 1 | 1.00 | 0.59 (0.49-0.70) | 0.51 (0.43-0.62) | 0.49 (0.40-0.59) | 0.26 (0.21-0.33) | <0.001 | 0.62 (0.57-0.66) |
| Model 2 | 1.00 | 0.73 (0.60-0.88) | 0.53 (0.43-0.66) | 0.43 (0.34-0.55) | 0.36 (0.26-0.48) | <0.001 | 0.64 (0.58-0.71) |
| Model 3 | 1.00 | 0.81 (0.67-0.97) | 0.64 (0.51-0.80) | 0.57 (0.45-0.74) | 0.53 (0.39-0.72) | <0.001 | 0.75 (0.67-0.84) |
| **Alzheimer’s disease** |  |  |  |  |  |  |  |
| **Cases/person-years** | 653/829,954 | 378/864,434 | 316/869,355 | 322/874,851 | 213/888,413 |  |  |
| Model 1 | 1.00 | 0.55 (0.48-0.62) | 0.45 (0.40-0.52) | 0.46 (0.40-0.53) | 0.30 (0.25-0.35) | <0.001 | 0.63 (0.60-0.67) |
| Model 2 | 1.00 | 0.80 (0.70-0.91) | 0.70 (0.60-0.83) | 0.69 (0.57-0.82) | 0.67 (0.54-0.84) | <0.001 | 0.83 (0.76-0.89) |
| Model 3 | 1.00 | 0.84 (0.73-0.96) | 0.77 (0.65-0.91) | 0.78 (0.65-0.94) | 0.80 (0.64-0.99) | 0.04 | 0.88 (0.82-0.96) |
| **FEV_1_/FVC** |  |  |  |  |  |  |  |
| **All-cause dementia** |  |  |  |  |  |  |  |
| **Cases/person-years** | 1,294/845,279 | 1,024/846,540 | 765/882,046 | 706/880,798 | 548/872,345 |  |  |
| Model 1 | 1.00 | 0.78 (0.72-0.85) | 0.56 (0.51-0.61) | 0.52 (0.47-0.57) | 0.40 (0.37-0.45) | <0.001 | 0.75 (0.73-0.77) |
| Model 2 | 1.00 | 0.95 (0.87-1.03) | 0.77 (0.71-0.85) | 0.84 (0.77-0.92) | 0.87 (0.79-0.97) | <0.001 | 0.91 (0.88-0.93) |
| Model 3 | 1.00 | 0.98 (0.90-1.06) | 0.80 (0.73-0.87) | 0.86 (0.78-0.94) | 0.89 (0.80-0.99) | <0.001 | 0.92 (0.89-0.94) |
| **Vascular dementia** |  |  |  |  |  |  |  |
| **Cases/person-years** | 323/845,279 | 224/846,540 | 162/882,046 | 145/880,798 | 104/872,345 |  |  |
| Model 1 | 1.00 | 0.69 (0.58-0.81) | 0.48 (0.39-0.57) | 0.43 (0.35-0.52) | 0.31 (0.25-0.38) | <0.001 | 0.72 (0.68-0.75) |
| Model 2 | 1.00 | 0.87 (0.73-1.03) | 0.69 (0.57-0.84) | 0.74 (0.60-0.90) | 0.72 (0.58-0.90) | <0.001 | 0.87 (0.83-0.92) |
| Model 3 | 1.00 | 0.91 (0.76-1.08) | 0.72 (0.59-0.87) | 0.74 (0.60-0.90) | 0.74 (0.59-0.92) | <0.001 | 0.89 (0.84-0.94) |
| **Alzheimer’s disease** |  |  |  |  |  |  |  |
| **Cases/person-years** | 540/845,279 | 469/846,540 | 342/882,046 | 292/880,798 | 239/872,345 |  |  |
| Model 1 | 1.00 | 0.86 (0.76-0.97) | 0.60 (0.52-0.69) | 0.51 (0.44-0.59) | 0.42 (0.36-0.49) | <0.001 | 0.75 (0.73-0.78) |
| Model 2 | 1.00 | 1.04 (0.92-1.18) | 0.83 (0.73-0.95) | 0.85 (0.73-0.98) | 0.94 (0.81-1.10) | 0.02 | 0.92 (0.88-0.95) |
| Model 3 | 1.00 | 1.06 (0.94-1.21) | 0.84 (0.73-0.97) | 0.85 (0.74-0.99) | 0.96 (0.82-1.13) | 0.047 | 0.92 (0.89-0.96) |

FVC: forced vital capacity; FEV_1_: forced expiratory volume in one second.

Model 1: crude.

Model 2: adjusted for age, sex, assessment centre, Townsend deprivation index, and education. Analysis of FVC was additionally adjusted for height.

Model 3: further adjusted for 12 potentially modifiable risk factors for dementia including education, hearing loss, traumatic brain injury, hypertension, alcohol >21 units per week, obesity, smoking, depression, social isolation, physical inactivity, air pollution, and diabetes. The model presented in the main manuscript includes APOE4 alleles as well.

**Supplementary** Table 8. Colocalisation results for lung function measures and cognitive traits using default prior probabilities.

| **Gene** | **LF trait** | **Cognitive trait** | **Highest PP** | **SNP** | **LD (R^2^)** |
| --- | --- | --- | --- | --- | --- |
| *CSNK2B* | FEV_1_/FVC | Fluid intelligence | H4: 0.986 | **rs9267531** | ― |
|  |  | Pairs matching | H4: 0.965 | “ | ― |
|  | FVC | Fluid intelligence | H4: 0.994^*^ | “ | ― |
|  |  | Pairs matching | H4: 0.988^*^ | “ | ― |
| *NFATC3* | FEV_1_/FVC | Fluid intelligence | H4: 0.860^*^ | **rs548092276 & rs11275011** | 0.85^§^ |
| *ITGAV* | FEV_1_/FVC | Pairs matching | H4: 0.794 | rs2084448^†^ | ― |
| *KAT8* | FVC | Pairs matching | H4: 0.994 | **rs138259061** | ― |
|  |  | AD^‡^ | H3: 0.622 | rs1978487, rs11865499^**^ | 0.23 |
| *PTCH1* | FVC | Reaction time | H3: 0.880^*^ | rs113154802, rs539078574 | 0.13 |
|  |  | Fluid intelligence | H3: 0.999^*^ | rs113154802, rs28496034 | 0.19 |
|  | FEV_1_/FVC | Reaction time | H4: 0.747^*^ | **rs2297086 & rs539078574** | 0.54 |
|  |  |  | H3: 0.881^*^ | rs75614054, rs539078574 | 0.13 |
|  |  | Fluid intelligence | H4: 0.653^*^ | **rs2297086 & rs28496034** | 0.86 |
|  |  |  | H3: 0.999^*^ | rs75614054, rs28496034 | 0.19 |
| *MMP24* | FVC | Symbol digit substitution | H4: 0.532 | **rs6120880** | ― |
| *TNS1* | FEV_1_/FVC | Vascular dementia | H4: 0.548 | rs2571445^††^ | ― |

Highlighted in bold are variants with evidence for colocalisation where the variants for each trait in the colocalisation pair were either the same (a high gene PP H4 and a high SNP PP H4: variants in *CSNK2B1*, *KAT8*, and *MMP24*, and *TNS1*) or distinct but with correlated signals (high SNP PP H4 for the pair by coloc-SuSiE: variants in *NFATC3* and *PTCH1* separated by ‘&’).

LF: Lung function, CF: Cognitive function, PP: Posterior probability, FVC: Forced vital capacity, FEV_1_: Forced expiratory volume in the first second, LD: Linkage disequilibrium.

^*^ By SuSiE-coloc

^§^ Variants rs548092276 and rs11275011 are not in 1000G reference panel (GRCh37 and GRCh38), so R^2^ is reported based on the two variants data in UKB.

^†^ SNP.PP.H4 = 18.8% (the highest reported for the variants in *ITGAV*)

^‡^ From GWAS meta-analysis

^**^Variants detected by fine mapping (the highest PP) as SuSiE did not operate for *KAT8*.

^††^ SNP.PP.H4 = 47.0% (the highest reported for the variants in *TNS1*)

**Supplementary** Table 9. Characteristics of variants and their effects where the lung function measure colocalised with cognitive traits or different variants were associated with each trait.

| **GENE** | **ID** | **Effect allele** | **MAF** | **Lung function** | | | **Neurocognitive** | | |
| --- | --- | --- | --- | --- | --- | --- | --- | --- | --- |
|  |  |  |  | **Trait** | **Beta** | **P** | **Trait** | **Beta** | **P** |
| *MMP24* | rs6120880 | C | 0.435 | FVC | 11.35 | 4.75E-14 | Symbol digit substitution | -0.023 | 9.28E-04 |
| *TNS1* | rs2571445 | A | 0.391 | FEV_1_/FVC | -0.112 | 2.77E-12 | Vascular Dementia | 0.112^¶^ | 2.26E-03 |

FVC: Forced vital capacity, FEV_1_: Forced expiratory volume in the first second.

^¶^ OR (95% CI): 1.12 (1.05-1.19)

**Supplementary** Table 10: Function and expression of genes with evidence of shared pathways for lung function and cognitive traits.

| **Gene** | **Full name and function**  **(Biological category)** | **Lung function measure** | **CF traits** | **Evidence on tissue expression^*^** | | | |
| --- | --- | --- | --- | --- | --- | --- | --- |
|  |  |  |  | **Lung** | | **Brain** | |
|  |  |  |  | **RNA** | **Protein** | **RNA** | **Protein^†^** |
| ***CSNK2B*** | Casein kinase 2β: Ubiquitous protein kinase that regulates metabolic pathways, signal transduction, transcription, translation, and replication (*Transcriptional regulators*) | FEV_1_/FVC & FVC | Fluid intelligence  Pairs matching | ++ | ++ | +++ | +++ |
| ***NFATC3*** | Nuclear factor of activated T cells 3: Encodes a member of the nuclear factors of activated T cells family of transcription factors (*Transcriptional regulators*) | FEV_1_/FVC | Fluid intelligence | + | NA | + | NA |
| *ITGAV* | Integrin subunit α V: Encodes a member of the integrin a chain family (*Extracellular matrix*) | FEV_1_/FVC | Pairs matching | ++ | ― | +++ | ++ |
| ***KAT8*** | Lysine acetyltransferase 8: Encodes a member of the MYST histone acetylase protein family; the encoded protein regulates gene transcription by influencing chromatin conformation (*Transcriptional regulators*) | FVC | Pairs matching | ++ | NA | +++ | NA |
| ***PTCH1*** | Patched 1: Encodes a member of the patched family of proteins and a component of the hedgehog signalling pathway (*Growth factors*) | FEV_1_/FVC | Fluid intelligence  Reaction time | + | ++ | ++ | +++ |
| ***MMP24*** | Matrix metallopeptidase 24: Encodes a member of the peptidase M10 family of MMPs (*Extracellular matrix*) | FVC | Symbol digit substitution | + | NA | +++ | +++ |
| *TNS1* | Tensin 1: Encodes for a protein that localises to focal adhesions and crosslinks actin filaments (*Cell-to-cell adhesion and cytoskeleton*; *Extracellular matrix*) | FEV_1_/FVC | Vascular dementia | +++ | ++ | ++ | ++ |
| *SERPINC1* | Serpin family C member 1: Encodes a plasma protease inhibitor and a member of the serpin superfamily | FVC | Reaction time | + | + | + | + |

Highlighted in bold are genes with colocalisation signals; *ITGAV* and *TNS1* distinct variants were linked to lung function and cognitive traits.

^*^ Evidence of gene expression (high: +++, medium: ++, low: +), RNA expression from Expression Atlas ([www.ebi.ac.uk/gxa/home](http://www.ebi.ac.uk/gxa/home)) and protein expression from The Human Protein Atlas ([www.proteinatlas.org/humanproteome/tissue](http://www.proteinatlas.org/humanproteome/tissue)) for lung and for brain tissues.

† For all genes with evidence protein expression, it was detected in cerebral cortex, cerebellum, basal ganglia (caudate), and hippocampus (particularly neuronal cells), except for TNS1 that protein expression was only detected in cerebral cortex and cerebellum.
